# Ferroptosis Induction and YAP Inhibition as New Therapeutic Targets in Gastrointestinal Stromal Tumors (GISTs)

**DOI:** 10.3390/cancers14205050

**Published:** 2022-10-15

**Authors:** Marine Delvaux, Perrine Hagué, Ligia Craciun, Agnieszka Wozniak, Pieter Demetter, Patrick Schöffski, Christophe Erneux, Jean-Marie Vanderwinden

**Affiliations:** 1 Laboratory of Neurophysiology, Faculty of Medicine, Université Libre de Bruxelles, 1070 Brussels, Belgium; 2 Department of Pathology, Institut Jules Bordet, Université Libre de Bruxelles, 1070 Brussels, Belgium; 3 Laboratory of Experimental Oncology, Department of Oncology, KU Leuven, 3000 Leuven, Belgium; 4 IRIBHM, Campus Erasme, Université Libre de Bruxelles, 1070 Bruxelles, Belgium

**Keywords:** GIST, YAP, verteporfin, ferroptosis, TFRC, lipid peroxidation, cell survival

## Abstract

**Simple Summary:**

Despite targeted therapy with tyrosine kinase inhibitors (TKIs), unresectable or metastatic human GISTs generally relapse under treatment. Thus, alternative therapeutic approaches are needed to overcome GIST resistance. Ferroptosis, a cell death driven by iron-dependent phospholipid peroxidation, is an emerging therapeutic approach in cancers. However, the potential of ferroptosis induction in GISTs remains largely unknown. This study shows that GIST cell lines are highly sensitive to the type II ferroptosis inducer, RSL3. We also provide evidence that inhibition of YAP by verteporfin (VP) promotes ferroptosis in GIST cells while, surprisingly, CA3 mediates ferroptosis independently of YAP inhibition. Finally, we highlight a positive correlation between the highly expressed transferrin receptor protein 1 (TFRC), GISTs with elevated mitotic counts or higher risk, and with YAP expression/activation in human GIST tissue microarrays (TMA). Taken together, our results suggest that induction of ferroptosis and/or modulation of YAP activity offer promising perspectives for GIST treatment.

**Abstract:**

GISTs are sarcomas of the gastrointestinal tract often associated with gain-of-function mutations in KIT or PDGFRA receptor genes. While most GISTs initially respond to tyrosine kinase inhibitors, relapses due to acquired resistance frequently occur. The induction of ferroptosis, an iron-dependent form of non-apoptotic cell death, emerged as a novel therapeutic approach in cancers and remains poorly characterized in GISTs. We studied hallmarks of ferroptosis, i.e., lipid peroxidation, iron and glutathione content, and GPX4 protein expression in imatinib-sensitive (GIST882) and -resistant (GIST48) GIST cell lines. GIST cells were highly sensitive to the induction of ferroptosis by RSL3, which was reversed by liproxstatin and deferoxamine. Lipid peroxidation and ferroptosis were mediated by VP and CA3 in GIST cells through a significant decrease in antioxidant defenses. Moreover, VP, but surprisingly not CA3, inhibited a series of target genes downstream of YAP in GIST cells. The ferroptosis marker TFRC was also investigated by immunohistochemistry in GIST tissue arrays. TFRC expression was observed in all samples. High TFRC expression was positively correlated with high-risk GISTs, elevated mitotic count, and YAP nuclear localization, reflecting YAP activation. This study highlights ferroptosis as a novel cell death mechanism in GISTs, and a potential therapeutic target to overcome resistance to tyrosine kinase inhibitors.

## 1. Introduction

Gastrointestinal stromal tumors (GISTs) are the most frequent sarcomas of the gastrointestinal tract and derive from the interstitial cells of Cajal (ICCs) or their precursors [1]. ICCs are key players in the motility of the gut as they act as a pacemaker of the muscularis propria and mediate the transduction of the enteric nervous system inputs [2,3]. Approximately 85% of GISTs are driven by somatic gain-of-function mutations of the gene encoding the tyrosine kinase receptor (RTK) KIT (CD117) [1,4]. Surgery is the only curative treatment for GISTs; however, for unresectable or metastatic disease, the tyrosine kinase inhibitor (TKI) imatinib (Gleevec^®®^, Glivec^®®^) is the first line of therapy [5,6]. Unfortunately, most patients ultimately progress due to primary or secondary mutations of *KIT* or *PDGFRA*. Second- and third-line TKIs (regorafenib, sunitinib, etc.) provide only limited benefits [7,8]. Therefore, the development of novel therapeutic strategies remains essential to improve patient outcome.

We previously reported the involvement of the transcriptional co-activator YAP in the survival of imatinib-sensitive (GIST882) and -resistant (GIST48) human GIST cell lines [9]. YAP is located downstream of the Hippo pathway, which responds to diverse stimuli including cell–cell interaction, cellular stress, extracellular signals, cell polarity, and mechanical signals [10,11]. In its active unphosphorylated state, YAP binds transcriptional cofactors such as TEA domain family members (TEADs), and activates a transcriptional program of genes involved in cell proliferation, survival, differentiation, and tumorigenesis [10,12,13]. The YAP inhibitor verteporfin (VP) elicited massive cytotoxicity in GIST882 and GIST48 cell lines [9]. Moreover, nuclear YAP immunoreactivity, indicative of YAP transcriptional activity, was observed in 71% of primary human GIST tissues, providing evidence of YAP involvement in GIST biology [9]. Recently, a series of links between the Hippo–YAP pathway and ferroptosis have been identified [14,15,16]. Ferroptosis is a non-apoptotic iron-dependent form of cell death characterized by an accumulation of lipid peroxidation that leads to membrane damage [17,18,19]. Ferroptosis is the result of an imbalance between oxidative damage and antioxidant defense [19,20]. Oxidative damage is either caused by an accumulation of radical oxygen species (ROS), mainly produced by the Fenton reaction which involves free ferrous iron and H_2_O_2_, or lipid peroxidation mediated by iron-containing lipoxygenases (LOX) [18,19,21]. Free ferrous iron is mainly represented by the labile iron pool (LIP), which is regulated by different proteins involved in iron metabolism, such as transferrin receptor 1 (TFRC), ferritin (FTH, FTL), and ferroportin (FPN1). The antioxidant defenses involve the axis between SLC7A11, glutathione (GSH), glutathione peroxidase 4 (GPX4), and the nuclear factor erythroid 2-related factor 2 (NRF2). SLC7A11 is one of the two subunits of the cystine–glutamate antiporter system X_c_^−^ that imports extracellular cystine into the cytoplasm, where it is used in the biosynthesis of GSH, the cofactor of the antioxidant enzyme GPX4. This later reduces hydroperoxide lipids and thus prevents ferroptosis [18,19,20].

As ferroptosis emerges as a promising approach for cancer therapy [18,22,23,24], it appeared as an attractive mechanism to explore in GISTs. Ferroptosis sensitivity has recently been reported in imatinib-sensitive GIST-T1 cells (*KIT* exon 11 mutation [25]) and imatinib-resistant GIST R8 cells [26], which have been established from parental GIST-T1 cells treated with increasing doses of imatinib [27]. However, so far, ferroptosis has not been investigated in imatinib-sensitive GIST882 (*KIT* exon 13 mutation) cells, or in imatinib-resistant GIST48 cells (*KIT* primary exon 11 and resistant exon 17 mutations), which originate from a GIST patient who had progressed under imatinib treatment.

Here, we studied the impact of the class II ferroptosis inducer (FIN), RSL3, a direct GPX4 inhibitor, on the viability and lipid peroxidation of GIST882 and GIST48 cells. We next used two YAP inhibitors and tested different hallmarks of ferroptosis in GIST882 and GIST48 cells. Finally, as cancer cells appear to be “addicted to iron” [28,29] and as the TFRC emerged as a new cancer marker [30], we tested the expression of the TFRC in human GIST tissues and explored possible correlations with clinicopathological criteria and YAP expression.

## 2. Materials and Methods

### 2.1. Ethics Statement

The collection and analysis of human tissue samples were conducted in line with ethical standards according to the Declaration of Helsinki, as well as national and international guidelines. The study was approved by the Institutional Medical Ethics Committees of Erasmus Hospital and Faculty of Medicine, Université Libre de Bruxelles (reference number P2016/316); the Ethical Committee of Institut Jules Bordet (reference number CE 2964), Brussels, Belgium; and the Medical Ethics Committee, UZ Leuven, Leuven, Belgium (reference number S66100) for their respective human materials.

### 2.2. Cell Lines and Drugs

The human GIST882 cell line [31] was kindly provided by Dr. Jonathan A. Fletcher, Harvard Medical School, Boston, MA, USA. Cells were cultured at 37 °C in DMEM (Gibco, CA, USA, Cat# 41965-062), supplemented with 10% (*v*/*v*) FBS, 2% (*v*/*v*) penicillin–streptomycin (Gibco, CA, USA, Cat# 15140-122). The human GIST48 cell line [32] was kindly provided by Dr. Ronald DeMatteo, Perelman School of Medicine, Philadelphia, PA, USA. Cells were cultured at 37 °C in RPMI-1640 (Gibco, CA, USA, Cat# 31870-074), supplemented with 10% (*v*/*v*) FBS, 1% (*v*/*v*) L-glutamine (Gibco, CA, USA, Cat# 35050-038), 1% (*v*/*v*) HEPES (Gibco, CA, USA, Cat# 15630-056), 0.1% (*v*/*v*) 2-mercaptoethanol (Gibco, CA, USA, Cat# 31350-010), and 0.5% (*v*/*v*) penicillin–streptomycin. Verteporfin and cumene hydroperoxide were purchased from Sigma, St. Louis, MO, USA (Cat# C0737, SML0534, and 247502, respectively). Erastin, liproxstatin-1, deferoxamine mesylate, RSL3, CA3, and staurosporine were purchased from Selleckchem, Houston, TX, USA (Cat# S7242, S7699, S5742, S8155, S8661, and S1421, respectively).

### 2.3. Cell Viability Assay

Cell viability was estimated using a WST-1 assay (Roche, Indianapolis, IN, USA, Cat# 5015944001). GIST882 and GIST48 cells were seeded in 96-well plates (TPP Techno Plastic Products AG, Trasadingen, Switzerland) at a concentration of 10,000 cells/well supplemented with 100 μL of medium 72 h before drug treatments. Drug concentrations were chosen based on previous studies [33,34,35,36]. After drug treatment, 10 μL of WST-1 reagent was added and plates were incubated at 37 °C for either 2 h or 3 h for GIST882 and GIST48 cells, respectively. Absorbance was measured at 450 nm on a plate reader (iMark Microplate Absorbance Reader, BioRad, Hercules, CA, USA). IC50s were calculated using Prism 7 software (GraphPad Software, Inc., La Jolla, CA, USA) and CompuSyn software (https://www.combosyn.com/, accessed on 18 August 2022)

### 2.4. Immunohistochemistry

Cohorts of archival GIST primary tumors and metastatic GISTs from formalin-fixed slides, paraffin-embedded (FFPE) material, and tissue microarrays (TMA) were obtained from the Laboratory of Pathological Anatomy, Jules Bordet Institute, Brussels, Belgium and from the Laboratory of Experimental Oncology, Department of Oncology, KU Leuven, Leuven, Belgium. Clinicopathological features are given in Tables S1–S3. The risk of relapse after the curative resection of primary GISTs was determined according to Miettinen’s classification [37]. FFPE slides and TMA were rehydrated through toluol and graded alcohol solutions, before then being heated at 90 °C in citrate buffer 0.01M (pH 6.0) antigen retrieval solution for 20 min to unmask the antigen. Slides were then cooled for 15 min before being put in a 0.1% H_2_O_2_–methanol solution for 30 min to block endogenous peroxidase. After washing, slides were incubated with a TBS-Triton X-100, 10% NHS blocking solution for 1 h, followed by the primary antibodies diluted in a TBS-Triton X-100 0.1% and 1% NHS solution overnight at RT in a humid chamber. Sections were rinsed and incubated with a secondary biotinylated antibody for 1 h followed by an ABC solution (ABC Kit Standard; Vector Laboratories, Burlingame, CA, USA, Cat# PK-6100) for 1 h. Revelations with nickel-enhanced DAB (DAB-Ni) were performed at RT for 5–10 min, resulting in a black precipitate. The DAB-Ni solution was prepared by dissolving 0.06 g of nickel ammonium sulfate (Fluka, Buchs, Switzerland, Cat# 09885) and 2 mg of DAB (Sigma-Aldrich, St. Louis, MO, USA, Cat# D5637) in 10 mL of 0.05 M Tris/HCl, pH 8. Before use, 1 μL of 30% H_2_O_2_ (Thermo Fisher Scientific, MA, United States, Cat# 241022500) was added to the DAB-Ni solution. Slides were dehydrated through graded alcohol and toluol solutions, mounted with DPX (Merck, NY, United States) and stored at room temperature. A list of antibodies is given in Table S4.

For each TMA, KIT, YAP, and TFRC, staining intensities were scored individually by two researchers (Jean-Marie Vanderwinden and Marine Delvaux) blinded for clinicopathological information, and the results were assembled by consensus. For Bordet TMA, ten replicate samples were available, while for KU Leuven TMA, two replicate samples were available. For KIT and YAP staining, samples were scored as negative (−), positive (+), or strongly positive (++). None of the samples were graded as negative for KIT expression; thus, the expression scoring was +/++. The YAP location was also evaluated and defined as nuclear (N) or diffuse (D) or mixed (M). Nuclear and mixed YAP corresponded to active YAP, while diffuse YAP was considered to be an inactive YAP. TFRC staining was graded as negative (−), weakly positive (+), moderately positive (++), and strongly positive (+++). None of the samples were graded as negative for TFRC expression; thus, the TFRC expression grading was +/++/+++.

### 2.5. Microscopy and Image Processing

Image acquisition and analysis were performed at LiMiF https://limif.ulb.be/, the Light Microscopy Facility, Université Libre de Bruxelles, Faculty of Medicine, Campus Erasme, Brussels. IHC slides were observed on a AxioObserver Z1 inverted microscope (Zeiss, Jena, Germany), using a Plan Apochromat 20x/0.8 dry objective (Zeiss). Transmitted light illumination was provided by a HAL100 halogen lamp and condenser in “bright-field position”. Images (1920 by 1216 pixels, pixel size (x-y): 0.293 micron by 0.293 micron) were acquired with an Axiocam 702 monochrome camera (Zeiss) as proprietary *.czi files. Files were processed with Zen 2.5 (Blue Edition) software (Zeiss). Images were displayed in the linear mode with manual contrast adjustment and exported as 16 bits uncompressed TIF files.

### 2.6. Real Time Quantitative PCR (qPCR)

The total RNA was extracted using the RNeasy Mini Kit (Qiagen, Valencia, CA, USA, Cat# 74104), according to the manufacturer’s instructions. Genomic DNA was removed using the RNase-Free DNase set (Qiagen, Valencia, CA, USA, Cat# 79274). Then, 1 µg of RNA was retrotranscripted into cDNA using LunaScript^®®^ RT SuperMix Kit (New England BioLab, Ipswich, MA, USA, Cat# E3010L), according to the manufacturer’s instructions. The cDNA reverse transcription products were amplified with specific primers (Table S5) by qPCR using SYBR Green chemistry on a QuantStudio™ 3 Real-Time PCR System (Applied Biosystems, Foster City, CA, USA). Identical thermal profile conditions, namely 95 °C for 10 min, followed by 40 cycles of 95 °C for 15 s and 60 °C for 1 min, were used for all primer sets. Emitted fluorescence was measured during the annealing–extension phase and amplification plots were generated using the sequence detection system.

Transcriptional quantification, relative to GAPDH and HPRT1 reference genes, was performed using qBase+ software (Biogazelle, Zwijnaarde, Belgium). Statistical analysis was performed with Prism 9 software (GraphPad Software, Inc., La Jolla, CA, USA), using the multiple-ratio paired t-test.

### 2.7. RNA Sequencing Analysis

RNA quality was checked with a fragment analyzer (Agilent Technologies). Indexed cDNA libraries were obtained using the TruSeq Stranded mRNA Sample Prep Kit (Illumina), following the manufacturer’s recommendations. The multiplexed libraries were loaded on a NovaSeq 6000 (Illumina) using a S2 flow cell and sequences were produced using a 200 Cycles Reagent Kit. Paired-end reads were mapped against the human reference genome GRCh38 using STAR_2.5.3a software to generate read alignments for each sample. Annotations of Homo_sapiens.GRCh38.90.gft were obtained from http://sftp.ensembl.org./ (accessed on 12 August 2022). After the transcripts were assembled, gene-level counts were obtained using HTSeq-0.9.1 and normalized to 20 million aligned reads. Differential expression analysis was performed using the libraries DESeq2 version 1.36.0 (The R Foundation for Statistical Computing, Vienna, Austria) in R software version 4.2.1 (The R Foundation for Statistical Computing, Vienna, Austria). All volcano plots showed results of RNAseq as the statistical significance (adjusted *p*-value or q-value) versus the log2 FC and were generated using the libraries from ggplot2 version 3.3.36 in R software version 4.2.1. Genes with an adjusted *p*-value (q-value) ≤ 0.01 and an absolute log 2-fold change (abs log2 FC) > 0.5 were considered to be statistically significant. The ferroptosis database [38] used for the analysis was retrieved on http://www.zhounan.org/ferrdb/ (accessed on 19 September 2022).

### 2.8. Western Blot Analysis

Cell samples were lysed for 2 h at 4 °C in extraction buffer 5X PTR, purchased from Abcam (ab193970, Cambridge, United Kingdom), supplemented with extraction enhancer buffer 50X (Abcam, Cambridge, United Kingdom, Cat# ab193971) and EDTA-free protease inhibitor complete™ (Roche, Indianapolis, IN, USA, Cat# 11873580001). The total protein concentration was measured based on the Bradford assay using the Bio-Rad Protein Assay Kit II (Bio-Rad, Hercules, CA, USA, Cat# 5000002). Proteins were denatured in Laemmli sample buffer (Bio-Rad, Hercules, CA, USA, Cat# 1610747), heated at 95 °C for 5 min, separated by SDS-PAGE on 12% or 15% polyacrylamide gel, and transferred on a 0.2 μm nitrocellulose membrane (Bio-Rad, Hercules, CA, USA, Cat# 1620168). Each membrane was blocked with Intercept blocking buffer PBS (Li-Cor, Lincoln, NE, USA, Cat# 927-70001). Primary antibodies raised in different species and secondary antibodies, coupled with different fluorochromes (800 Li-Cor and 680 Li-Cor), were sequentially imaged on an Azure c500 imaging system (Azure Biosystems, Dublin, CA, USA) and displayed in green and red, respectively. Image Lite Studio quantification software (version 5.2.5, LI-COR Biosciences, Lincoln, NE, USA) was used to quantify the fluorescence signals. A list of antibodies used is given in Table S6. All the whole western blot figures can be found in the Supplementary Materials.

### 2.9. GSH Measurements

Glutathione contents were measured using Glutathione Colorimetric Assay Kit (BioVision, CA, USA, Cat# K261). GIST882 cells were seeded at 500,000 cells/well and GIST48 cells at 300,000 cell/well in 6-well plates. Cells were treated with DMSO, CA3 (0.670 µM), or VP (2 µM for GIST882 cells and 0.5 µM for GIST48 cells) for 24 h and then harvested. Cell pellets were washed twice in ice-cold 0.01M PBS pH 7.4 and then lysed in ice-cold glutathione buffer for 20 min on ice. Cell lysates were centrifuged for 10 min at 13,000 rpm at 4 °C and each supernatant was transferred to a new tube. A small volume from the supernatant was taken for protein quantification. Next, 5% sulfosalicylic acid (SSA) was added to the supernatant to precipitate and remove proteins from the sample and to protect GSH oxidation. Each sample was then centrifuged at 8000× *g* for 10 min at 4 °C and the supernatant was transferred to a new tube for the next steps, according to the instructions in the kit. The absorbance was read at 415 nm on a plate reader (iMark Microplate Absorbance Reader, BioRad, Hercules, CA, USA). The content of GSH of each sample was calculated using a standard curve and normalized to the protein content in the lysate (µg GSH/mg protein). Results are shown as scatter plots and are expressed as means ± SD of fold changes (treated samples normalized to untreated cells).

### 2.10. Ferrous Iron Measurements

Ferrous iron was measured using the colorimetric QuantiChrom Iron Assay Kit (BioAssay Systems, CA, Cat# DIFE-250). Briefly, cells were seeded in six-well plates (500,000 GIST882 cells or 300,000 GIST48 cells per well) 72 h before the start of the assay. Cells were treated for 24 h with VP or CA3 using concentrations mentioned above. The next day, cells were washed with PBS and lysed on ice for 20 min in 55 µL of lysis buffer containing 1% Triton X-100 (PBS + 1% Triton X-100 + cOmplete^™^ EDTA-free protease inhibitor cocktail (Roche, Cat# 11873580001)). Cell lysates were centrifuged at 4 °C for 15 min at 13,000 rpm, and each supernatant was transferred to a new 1.5 mL tube. From the supernatant, 2 µL was taken aside for protein quantification and the remaining material was used for ferrous iron measurements following instructions of the kit. The absorbance was read at 595 nm on a plate reader (iMark Microplate Absorbance Reader, BioRad, Hercules, CA, USA). Ferrous iron concentration was calculated using a standard curve and normalized to the protein content of the lysate (µM/mg protein) for each sample. Results are shown as scatter plots and are expressed as means ± SD of fold changes (treated samples normalized to untreated cells).

### 2.11. Flow Cytometry

#### 2.11.1. Lipid Peroxidation

Flow cytometry analysis of C11-BODIPY green fluorescence was used as a readout for membrane lipid peroxidation, a hallmark of ferroptosis. When C11-BODIPY ^581/591^ is oxidized, its emission fluorescence shifts from 591 nm (red) to 510 nm (green) [39]. Cumene hydroperoxide (CumOOH), a strong oxidant, was used as a positive control for lipid peroxidation. Cells were seeded in a 6-well plate (TPP Techno Plastic Products AG, Trasadingen, Switzerland) at a cell density of 300,000 or 500,000 per well for GIST48 and GIST882 cells, respectively, supplemented with 2 mL of medium. After drug treatment, 1 µM of BODIPY™ 581/591 C11 (Invitrogen, OR, USA, Cat# D3861) was added to the well and incubated at 37 °C for 30 min. Cells were treated with either 50 µM of CumOOH for 45 min (GIST882 cells) or 100 µM for 30 min (GIST48) (Figures S1 and S2). Cells were washed twice with PBS 1x (Gibco^TM^, CA, USA, Cat# 10010-056) and trypsinized in trypsin-EDTA (0.25%) (Gibco^TM^, CA, USA, Cat# 25200-056). Cells were harvested and centrifuged for 2 min at 3000 rpm. The pellet was resuspended in 500 µL of cold PBS/FBS 2%. Hoechst 33342 (Invitrogen, OR, USA, Cat# H3570) was added prior to flow cytometry to exclude dead cells. Flow cytometry was performed using a BD LSRFortessa™ X-20 cell analyzer (BD Bioscience). Green fluorescence was detected using a 488 nm wavelength laser with a 505 nm dichroic mirror and a band-pass emission filter (530/30 nm), and blue fluorescence was detected using a 405 nm wavelength laser with a band-pass emission filter (450/50 nm).

#### 2.11.2. Caspase-3 Active Staining

Apoptosis was detected by flow cytometry using the FITC Active Caspase-3 Apoptosis Kit (BD Biosciences Pharmingen, SanDiego, CA and Lexington, KY, USA, Cat# 550480). Briefly, GIST48 cells were seeded in 6-well plates at a cell density of 300,000 cells/ well. Cells were treated with 1 µM of staurosporine (STS) as a positive control for 4 h or with CA3 (0.670 µM) or VP (0.5 µM) for 24 h. Cells were collected, centrifuged, and resuspended in PBS. Then, 250,000 cells per test were transferred to a new vial and then centrifuged. Supernatants were removed and 100 µL of BD cytofix–cytoperm was added to each pellet for 30 min on ice. Cells were washed with BD Perm/Wash buffer (1x) and pelleted. Supernatants were removed and caspase-3 active antibody was added to each tube at a concentration of 1/15 (diluted in BD Perm/Wash buffer 1x) for an incubation time of 1 h at room temperature. Cells were washed with BD Perm/Wash buffer (1x), pelleted, resuspended in PBS/FBS 2%, and analyzed by flow cytometry. Flow cytometry was performed using a BD LSRFortessa™ X-20 cell analyzer (BD Bioscience). Green fluorescence was detected using a 488 nm wavelength laser with a 505 nm dichroic mirror and a band-pass emission filter (530/30 nm).

#### 2.11.3. Flow Cytometry Analysis

Data from the flow cytometry experiment were saved in the proprietary *.fsc file format. Analysis was performed using FlowJo 10.5.3 software (BD Biosciences, NJ, USA). Cells were selected for granularity (SSC-A x FSC-A) and singlets (FSC-W x FSC-H). Living cells were selected (Hoechst x FSC-A) and represented as a FITC-A histogram. Data were exported as CSV channel values. On the upper panel, Cumming estimation plots display all data points, presented as a swarm plot, and their distribution [40]. The number of events (single cells) analyzed is indicated in the figure. The size effect (mean difference) is presented as a bootstrap sampling distribution and depicted as a dot with a 95% confidence interval (95% CI), indicated by the ends of the vertical error bars on the lower axis (Figures S1 and S2). The mean difference in green fluorescence intensity of treated cells compared to untreated cells (DMSO) for multiple independent experimental replicates are shown as scatter plots ± SD, generated using Prism 9 software (GraphPad Software, Inc., La Jolla, CA, USA).

### 2.12. Statistics

Estimation statistics were used for flow cytometry analysis to focus on the magnitude of the effect (effect size) and its precision. “Data Analysis by Estimation Statistics” (DABEST) software [40] (https://www.estimationstats.com/#/ (accessed on 22 July 2022)) was used as a Python script (https://github.com/ACCLAB/DABEST-python, accessed on 22 July 2022), running on a local PC to generate Cumming plots, for experiments that share one reference control group. For all other experiment, statistical analysis was performed with Prism 9 software (GraphPad Software, Inc., La Jolla, CA, USA), using ordinary one-way ANOVA, followed by Tukey’s test for ferrous iron and GSH measurements, the Pearson normality test, repeated-measures one-way ANOVA with Geisser–Greenhouse correction and Tukey’s multiple comparisons test for Western blot experiments, and multiple-ratio paired t-test for qPCR. Contingency tables and Freeman–Halton extensions of Fisher’s test were performed to study the relationship between TFRC expression and non-continuous variables, such as tumor location, sex, mitotic figures, histological type, risk classification, YAP-ir, YAP activation, and KIT-ir. The Kruskal–Wallis test was used to compare the mean of tumor size between the TFRC+++, TFRC++, and TFRC+ GIST samples. A *p*-value smaller than 0.05 was regarded as statistically significant for iron and GSH measurements and for Western blot quantifications. For the qPCR experiment, and for biological relevance, a higher stringency was applied and a *p*-value smaller 0.01 was regarded as statistically significant. All data are presented as mean ± SD.

### 2.13. Figures

Figures were prepared with Adobe Illustrator (Version 26.3.1, Adobe Inc., San Jose, CA, USA).

## 3. Results

### 3.1. RSL3, a Potent Ferroptosis Inducer in GIST882 and GIST48 Human Cell Lines

**Figure 1:**
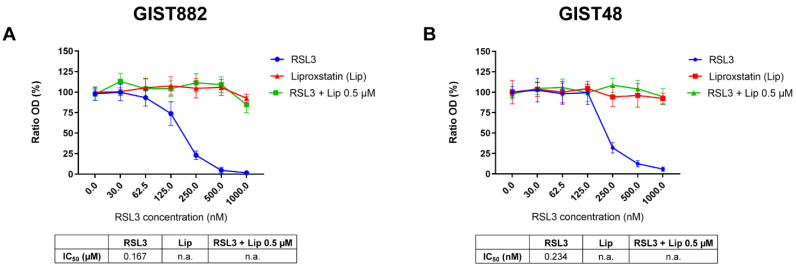
The class II ferroptosis inducer RSL3 drastically reduces GIST882 and GIST48 cell viability, which is completely rescued by the antioxidant liproxstatin. (**A**) WST-1 viability assays in GIST882 and (**B**) in GIST48 cells. Cells were treated for 3 h with RSL3 (0 to 1000 nM), liproxstatin (Lip) (0 to 2000 nM), or with a combination of different concentrations of RSL3 (0 to 1000 nM) and 0.5 µM of Lip. IC50 values are shown in tables under the viability graphs. Mean values ± SD are shown for three independent experiments with three technical replicates for each experiment.

Imatinib-sensitive (GIST882) and imatinib-resistant (GIST48) cells were treated with class II FIN RSL3, a direct GPX4 inhibitor for 3 h. We first assessed the cell viability. RSL3 drastically reduced GIST882 and GIST48 cell viability (Figure 1A,B). Liproxstatin (Lip), an antioxidant and ferroptosis inhibitor, protected GIST882 and GIST48 cells from RSL3 viability reduction.

**Figure 2:**
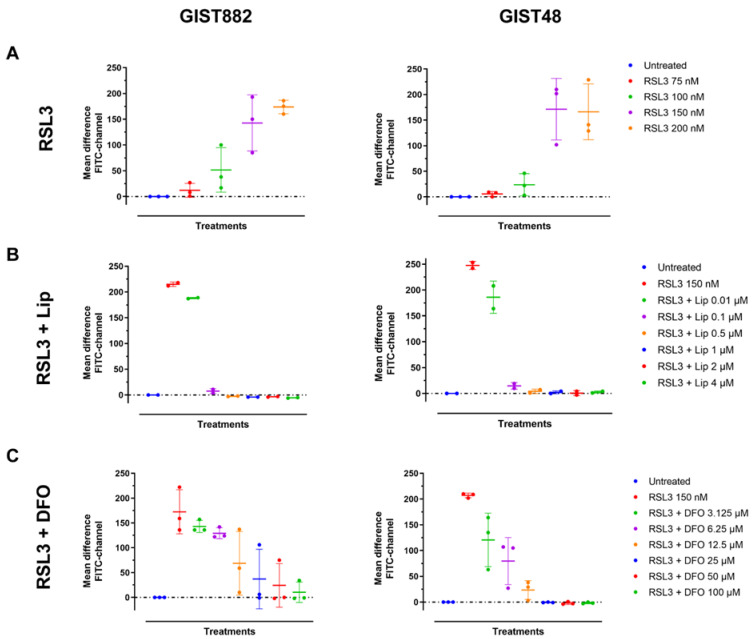
RSL3 treatment in GIST882 and GIST48 cells leads to lipid peroxidation, which is completely inhibited by the antioxidant Lip and by the iron chelator DFO. Lipid peroxidation was assessed by flow cytometry using C11-BODIPY581/591. GIST882 and GIST48 cells are shown in the left and right panels, respectively. (**A**) Cells were treated with indicated concentrations of RSL3 for 3 h and (**B**) with combinations of RSL3 150 nM and different concentrations of Lip or (**C**) different concentrations of DFO. Data were analyzed with DABEST software and scatter plots were generated using Prism 9 software. Data are presented as mean values ± SD. Mean values from two or three independent experiments.

We next investigated lipid peroxidation, the main hallmark of ferroptosis by flow cytometry using C11-BODIPY 581/591. RSL3 increased the mean difference in green fluorescence intensity (FITC-channel) after 3 h in a dose-dependent manner in both GIST882 and GIST48 cells (Figure 2A). The antioxidant Lip (from 0.1 µM to 4 µM) completely abolished lipid peroxidation induced by RSL3 (150 nM) in both GIST882 and GIST48 cells (Figure 2B). Deferoxamine (DFO), another ferroptosis inhibitor, acting through the chelation of iron, also prevented lipid peroxidation in both GIST882 and GIST48 cells (Figure 2C). We conclude that RSL3 is a potent ferroptosis inducer in both GIST882 and GIST48 cells.

### 3.2. DFO Protection against Viability Reduction Induced by VP in GIST882 and GIST48 Cells

**Figure 3:**
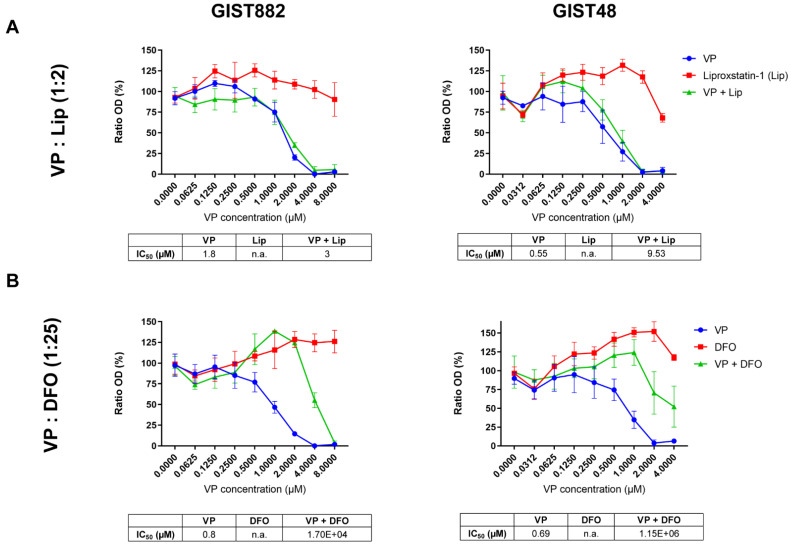
VP induces a massive loss of cell viability, which is reduced in the presence of DFO in both GIST882 and GIST48 cell lines. WST-1 viability assays in GIST882 and GIST48 are shown in the left and right panels, respectively. (**A**) Cells were treated for 24 h with a combination of different concentrations of VP and liproxstatin (Lip), an antioxidant, with a 1:2 ratio, or (**B**) with a combination of different concentrations of VP and DFO, an iron chelator, with a 1:25 ratio. IC50 values are shown in tables under the viability graphs. Mean values ± SD are shown for three or four independent experiments with three technical replicates for each experiment.

To investigate a potential link between ferroptosis and YAP, cell viability was assessed in GIST882 and GIST48 cells after 24 h of treatment with the combination of VP, an inhibitor of YAP [41,42], and the two ferroptosis inhibitors, Lip and DFO. We observed that Lip had a minor effect on viability reduction induced by VP in GIST882 and GIST48 cells, with a slight increase in IC50 values compared to the VP alone (Figure 3A). Interestingly, DFO increased the viability of VP-treated GIST882 and GIST48 cells, as reflected by higher IC50 values (Figure 3B). These data indicate that ferroptosis plays a role in VP-induced cell death and that iron is important in this mechanism.

### 3.3. The Protection of Ferroptosis Inhibitors against a Loss of Viability Mediated by CA3 in GIST882 and GIST48 Cells

**Figure 4:**
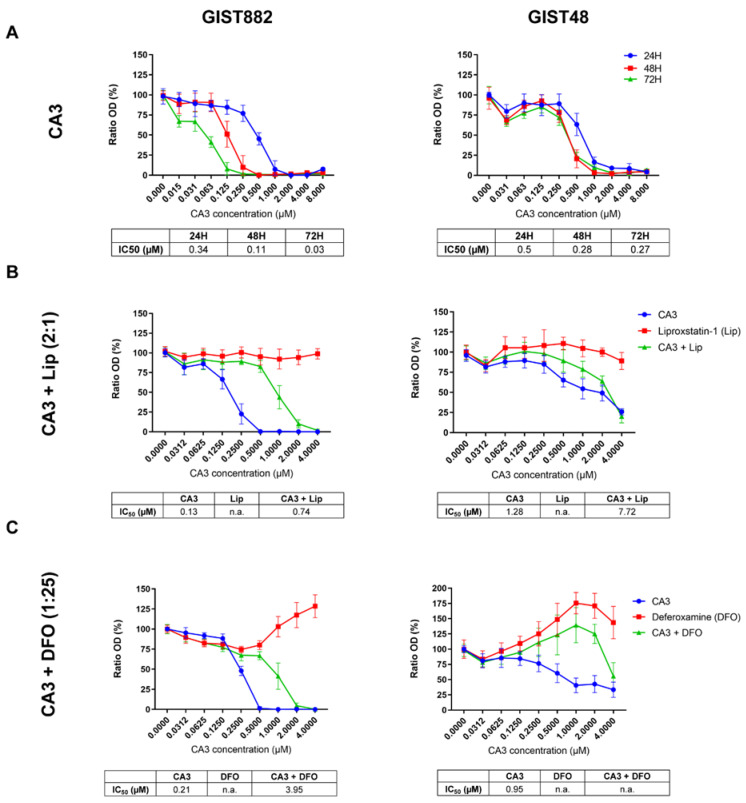
CA3 induces massive loss of cell viability, which is reduced by the iron chelator DFO and, to a lesser extent, by the antioxidant Lip in GIST882 and GIST48 cell lines. WST-1 viability assays in GIST882 and GIST48 cells are shown in the left and right panels, respectively. (**A**) Cells were treated with different concentrations of CA3 for 24, 48, and 72 h. (**B**) Cells were treated for 24 h with a combination of different concentrations of CA3 and Lip, with a 2:1 ratio, or (**C**) with a combination of different concentrations of CA3 and DFO, with a 1:25 ratio. IC50 values are shown under the viability graphs. Mean values ± SD are shown for three or four independent experiments with three technical replicates for each experiment.

VP is a photosensitizer used to treat macular degeneration [43] and has been shown to inhibit YAP-TEAD interaction independently of light [41,42]. However, ambient light can induce VP cytotoxicity through the formation of protein cross-linked oligomers and high-molecular-weight complexes [44] or by ROS production, as suggested in the patient-derived xenograft of acute lymphoblastic leukemia [45]. Although all experiments with VP were sheltered from ambient light to minimize possible photoactivation, we also tested CA3, a compound with a chemical structure distinct from VP, recently coined as a YAP inhibitor [46]. To investigate the effect of CA3 on GIST882 and GIST48 cell viability after 24, 48, and 72 h, we performed viability assays (Figure 4A). CA3 drastically reduced viability in both cell lines at 24, 48, and 72 h, as shown by their respective IC50 values. We then tested whether the antioxidant Lip and the iron chelator DFO reversed a reduction in viability induced by CA3 in GIST882 and GIST48 after 24 h (Figure 4B,C). Both Lip and DFO increased IC50 values when combined with CA3, as compared to CA3 alone. However, DFO was a more potent ferroptosis inhibitor than Lip to reverse the effect of CA3 on viability reduction. These data suggest that ferroptosis is the major cell death mechanism induced by CA3 in GIST882 and GIST48 cells.

### 3.4. The Induction of Lipid Peroxidation by VP and CA3, in GIST882 and GIST48 Cells

**Figure 5:**
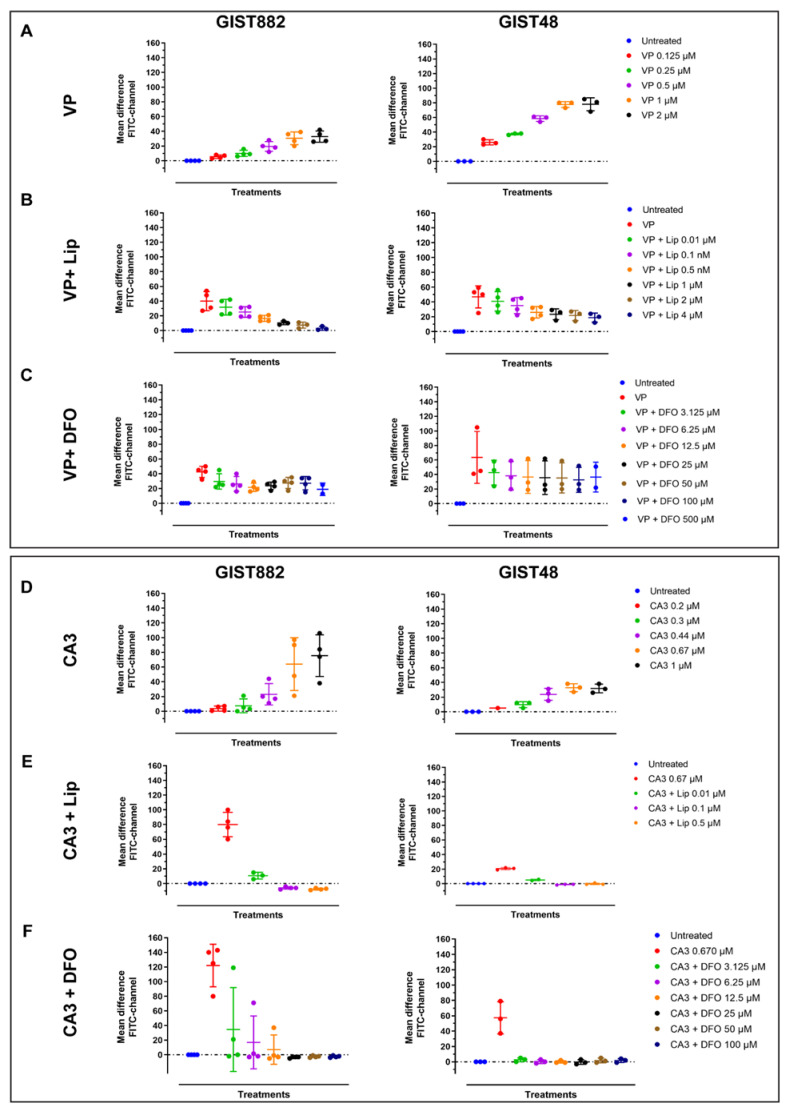
VP and CA3 trigger lipid peroxidation in GIST882 and GIST48 cell lines. Lipid peroxidation was assessed by flow cytometry. GIST882 and GIST48 cells are shown in the left and right panels, respectively. Upper panels: (**A**) GIST882 and GIST48 cells were treated with different concentrations of VP for 24 h. (**B**) Different concentrations of Lip were added to 2 µM of VP in GIST882 cells or 0.5 µM of VP in GIST48 cells. (**C**) Different concentrations of DFO were added to 2 µM of VP in GIST882 cells or 0.5 µM of VP in GIST48 cells. Lower panels: (**D**) GIST882 and GIST48 cells were treated with different concentrations of CA3. (**E**) Different concentrations of Lip were added to 670 nM of CA3 in GIST882 and GIST48 cells. (**F**) Different concentrations of DFO were added to 670 nM of CA3 in GIST882 and GIST48 cells. Data were analyzed with DABEST software and scatter plots were generated using Prism 9 software. Data are presented as mean values ± SD. Mean values from three or four independent experiments.

We next assessed lipid peroxidation in both GIST882 and GIST48 cells (Figure 5, left and right panels). We observed that VP increased the mean difference in green intensity fluorescence (FITC-channel) in GIST882 and GIST48 cells (Figure 5A) in a dose-dependent manner, corresponding to an increase in lipid peroxidation. The addition of different Lip and DFO concentrations halved the mean differences in green intensity fluorescence in GIST882 (Figure 5B,C, left panels) and GIST48 (Figure 5B,C, right panels). We next tested lipid peroxidation in GIST882 and GIST48 cells treated by CA3 for 24 h. CA3 also induced lipid peroxidation in a dose-dependent manner in GIST882 and GIST48 cells (Figure 5D). The antioxidant Lip completely abolished lipid peroxidation in GIST882 and GIST48 cells (Figure 5E). The iron chelator DFO also prevented GIST882 and GIST48 from lipid peroxidation induced by CA3 (Figure 5F). These data suggest that VP largely, albeit not exclusively, induces cell death, whereas CA3 seems to exert its cytotoxicity entirely through ferroptosis. We therefore estimated apoptosis in GIST48 after treatment with staurosporine (STS), used as positive control, and with VP or CA3. In GIST48, neither VP nor CA3 induced apoptosis after 24 h of treatment, whereas STS strongly induced apoptosis after 4 h (Figure S3A). In GIST882, we were unable to detect any activated caspase-3 in GIST882 cells, even in STS-treated cells (Figure S3B–E).

### 3.5. The Modulation of Ferrous Iron and Glutathione Contents by VP and CA3 Treatment in GIST882 and GIST48 Cells

**Figure 6:**
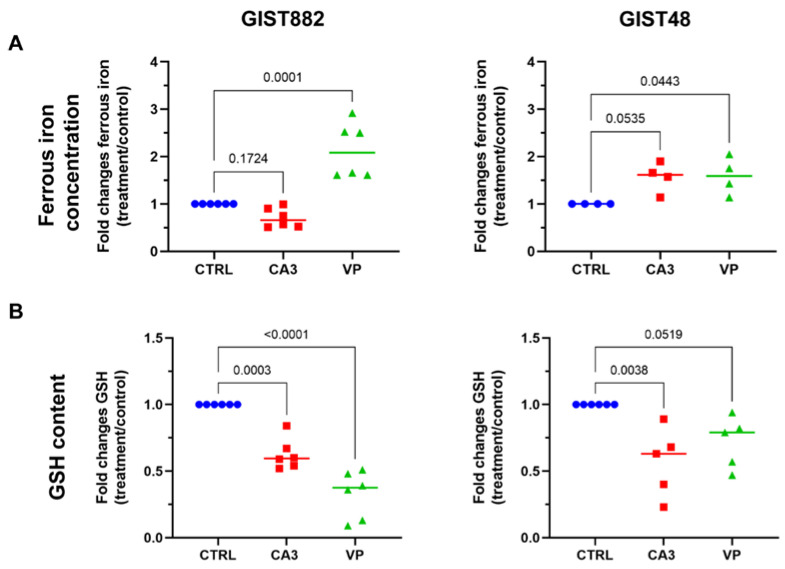
The effects of CA3 and VP on ferrous iron concentration and GSH content in GIST882 and GIST48 cells. GIST882 and GIST48 are shown in the left and right panels, respectively. (**A**) Ferrous iron concentration was determined in CA3- and VP-treated GIST882 and GIST48 cells. (**B**) GSH content was measured in CA3- and VP-treated GIST882 and GIST48 cells. Mean values ± SD are shown from four or six independent experiments. Statistical analysis was performed using one-way ANOVA, followed by Tukey’s test.

To understand how VP and CA3 induce lipid peroxidation-mediated ferroptosis in GIST cell lines, ferrous iron and glutathione (GSH) concentrations were measured. VP significantly increased ferrous iron concentration in both GIST882 and GIST48 cells after 24 h. CA3 exhibited only a minor non-significant increase in ferrous iron in GIST48 cells, but not in GIST882 cells (Figure 6A). GSH is another key regulator of ferroptosis and its content could be reduced following ferroptosis induction. We measured the GSH content after 24 h of treatment with VP or CA3 in both GIST882 and GIST48 (Figure 6B). VP treatment induced a significant depletion in the GSH content in GIST882 and a tendency towards a reduction in GIST48 cells. Interestingly, while CA3 failed to significantly increase ferrous iron content, it significantly reduced the GSH content in both cell lines. We conclude that VP acts on both iron and GSH metabolism, whereas CA3 mainly acts on antioxidant features of the cell through GSH metabolism.

### 3.6. Alteration of Gene Expression Induced by VP and CA3 Treatment

**Figure 7:**
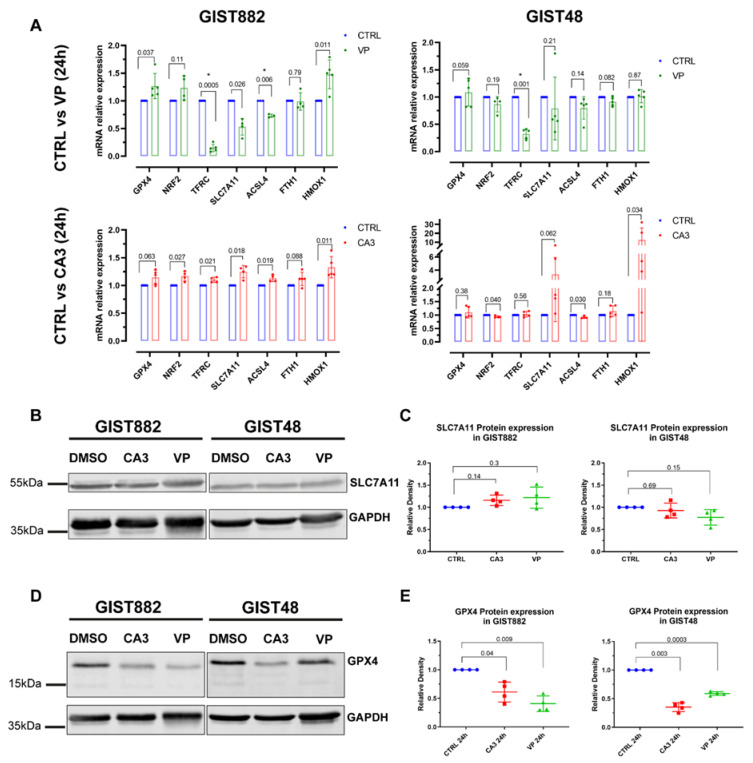
VP and CA3 do not modify the mRNA expression of a series of key ferroptosis genes but significantly deplete GPX4 protein expression in GIST882 and GIST48 cells. GIST882 and GIST48 cells were treated with VP (2 µM or 0.5 µM for GIST882 and GIST48, respectively) or CA3 (670 nM for GIST882 and GIST48) for 24 h. (**A**) The relative mRNA expression of indicated ferroptosis genes was analyzed by qPCR and presented as fold changes (treatment relative to control cells). *p*-values were calculated using the multiple-ratio paired t-test. *p*-values < 0.01 were considered statistically significant. Data presented as mean values ± SD are shown from four or five independent experiments with technical triplicates for each experiment. (**B**) SLC7A11 protein expression was determined by Western blot in GIST882 and GIST48 after CA3 and VP treatment and GAPDH was used as a loading control. (**C**) The quantification of SLC7A11 protein expression normalized to GAPDH and untreated cells (left panel: GIST882; right panel: GIST48). (**D**) GPX4 protein expression was determined by Western blot in GIST882 and GIST48 after CA3 and VP treatment and GAPDH was used as a loading control. (**E**) The quantification of GPX4 protein expression normalized to GAPDH and untreated cells (left panel: GIST882; right panel: GIST48). Thirty microgram protein added to each lane. Data presented as mean values ± SD. *p*-values were calculated with the Pearson normality test followed by repeated-measures one-way ANOVA with Geisser–Greenhouse correction and Tukey’s multiple comparisons test. *p*-values < 0.05 were considered statistically significant. All the whole western blot figures can be found in the supplementary materials.

The expression of a series of key ferroptotic genes (i.e., GPX4, NRF2, TFRC, SLC7A11 ACSL4, FTH1, and HMOX1), known to be affected following ferroptosis induction [18], was analyzed (Figure 7A). The results showed that VP reduced the expression of TFRC mRNA in both GIST882 and GIST48 cells after 24 h of treatment (Figure 7A, upper panels). The expression of other genes related to ferroptosis was not altered (Figure 7A, upper panels). We could not detect any significant modifications in the mRNA expression of all ferroptosis-related tested genes after 24 h of CA3 treatment (Figure 7A, lower panels). We extended the analysis by RNA sequencing (Figures S4 and S5). The differentially expressed genes (DEGs) identified after 24 h of treatment with either VP or CA3 were compared with the FerrDb database composed of genes known to promote (drivers) and prevent (suppressors) ferroptosis (http://www.zhounan.org/ferrdb, accessed on 19 September 2022) [38]. Multidimensional scaling (MDS) plots showed that, except for VP-treated GIST882 cells, CA3-treated GIST882 and GIST48 cells and VP-treated GIST48 cells were not well separated from control cells. The DEGs between untreated and VP-treated GIST882 cells were compared to the FerrDb database. It appeared that some ferroptosis suppressors were statistically downregulated (i.e., LAMP2, ACSL,3 and ENPP2) and two drivers were upregulated (i.e., CHAC1) (Figure S4C,D). We also confirmed by qPCR that TFRC mRNA was downregulated in VP-treated GIST882 cells. Except for the ENPP2 gene that was also downregulated in VP-treated GIST48 cells, the other ferroptosis-related DEG genes were not found in the other conditions, suggesting that VP and CA3 do not induce ferroptosis through major common changes in the expression of ferroptosis-related genes.

The mRNA expression of several genes downstream of YAP, CTGF, CYR61, and AMOTL2 was also analyzed by qPCR and RNA sequencing (Figure S6). While VP reduced, as expected for YAP inhibition, their expression in GIST882 and GIST48 cells, CA3 treatment, surprisingly, induced their up-regulation, rather than downregulation, in GIST882 cells, while no modification was observed in GIST48 cells.

### 3.7. The Strong Depletion in GPX4 Protein Expression Induced by VP and CA3 Treatment

We then questioned whether VP or CA3 modulate the expression of key proteins important for ferroptosis induction. We assessed SLC7A11 and GPX4 protein expression. Despite a reduction in the GSH content in both GIST882 and GIST48 by either VP or CA3 treatment, the SLC7A11 protein level was not affected in the two cell lines (Figure 7B,C). Conversely, a significant decrease in the GPX4 protein level was observed in both VP- and CA3-treated GIST882 and GIST48 cells compared to untreated cells, although GPX4 mRNA was not affected (Figure 7D,E). These data provide a common link between VP and CA3, as well as a downregulation of the GPX4 protein, a key enzyme which prevents lipid peroxidation and ferroptosis.

### 3.8. The Expression of the TFRC in Primary Human GIST Tissues and Correlation with Mitotic Counts, Risk Classification, and YAP Expression and Activation

**Figure 8:**
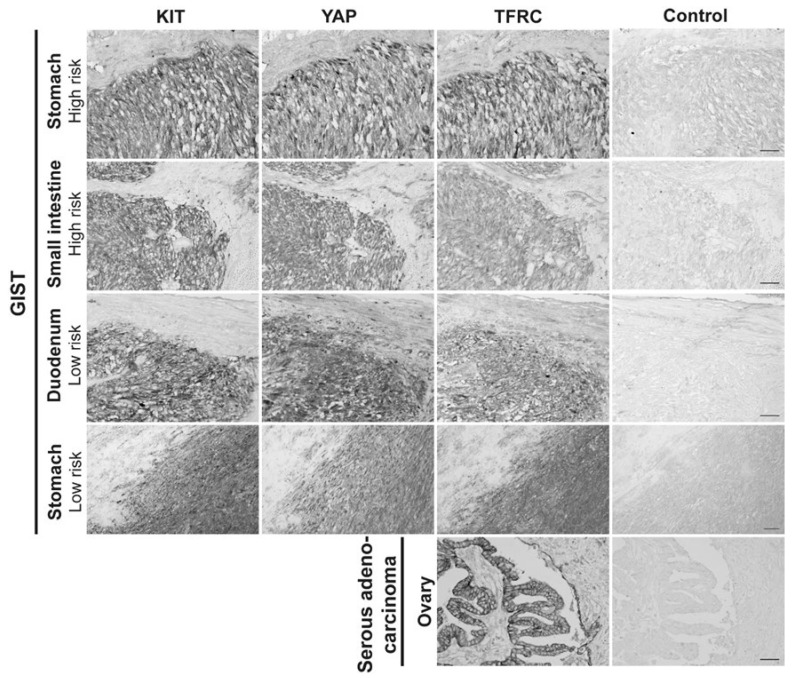
TFRC expression in human primary GISTs. Immunohistochemistry for KIT-ir, YAP-ir, and TFRC-ir in four different human primary GISTs located in the stomach and small intestine, with high or low risk of malignity. Ovarian serous adenocarcinoma was used as a positive control for TFRC-ir. Scale bar = 50 µm.

As the TFRC is described as a new cancer marker [30], the expression of the TFRC in human GIST tissues was assessed. IHC was performed on ovarian cancer tissue, used as a positive control [47] to validate TFRC antibody immunoreactivity (-ir), and on five primary GIST tissues with different clinicopathological features (Table S1). The TFRC-ir was observed in KIT- and YAP-positive regions of all GIST tissues tested (Figure 8). IHC was next performed on GIST tissue microarrays (TMAs) to increase the cohort and assess a potential correlation between the TFRC expression level and clinicopathological criteria, and/or YAP expression/activation.

**Figure 9:**
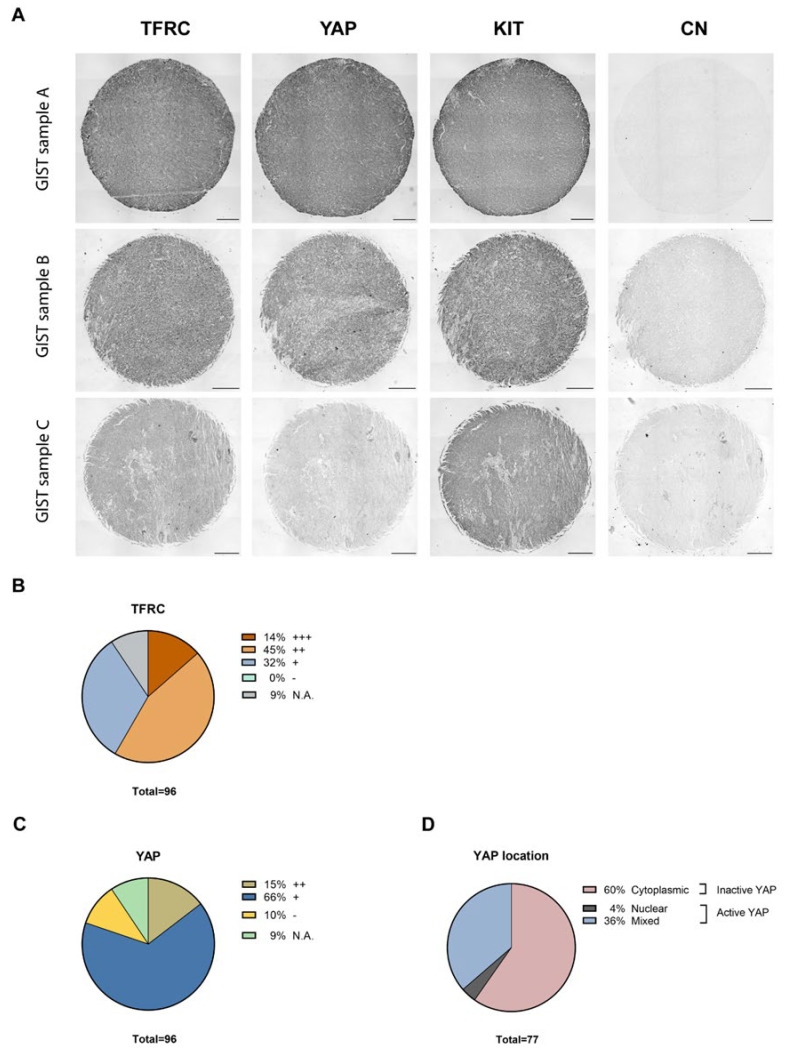
TFRC, YAP, and KIT immunohistochemistry on GIST tissue arrays. (**A**) Representative examples of TFRC, YAP, and KIT immunohistochemical staining in GIST TMA samples, graded as strong (+++), moderate (++), and weak (+) positive for TFRC-ir, and as strong positive (++) or positive (+) for YAP and KIT staining. Negative staining (−) was also observed for YAP-ir. Scale bars = 200 µm. (**B**) Proportions of GIST samples exhibiting different expression levels of the TFRC. (**C**) Proportions of GIST samples exhibiting different expression levels of YAP. (**D**) Proportions of GIST samples exhibiting active or inactive YAP. (N.A., Not Applicable).

Our cohort included a total of 96 GIST patients, with 9 being non-contributive because of poor tissue quality or loss during processing. GIST samples were stained for three markers, KIT, YAP, and TFRC (Figure 9A). All tissues displayed strong positive (++) or positive (+) KIT-ir, and TFRC-ir was also observed in all GIST specimens with a strong expression (+++) in 14% of the cohort, a moderate expression (++) in 45% of the cohort, and a weak expression (+) in 32% of the cohort (Figure 9B). YAP-ir was strongly positive (++) in 15% of GISTs, positive (+) in 66% of GISTs, and negative (−) in 10% of GISTs (Figure 9C). Finally, 60% of GIST samples displayed cytoplasmic YAP-ir, suggesting that YAP is inactive, whereas 40% displayed an active YAP (4% and 36% of nuclear and mixed location YAP-ir, respectively) (Figure 9D).

**Figure 10:**
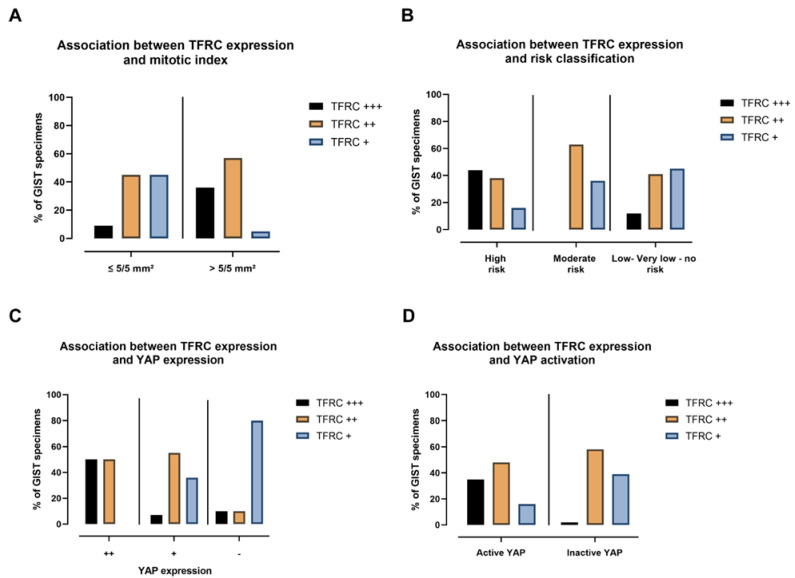
TFRC expression is associated with mitotic index, risk classification, YAP expression, and activation. (**A**) The percentage of TFRC-ir associated with mitotic index, (**B**) risk classification, (**C**) YAP-ir, and (**D**) YAP activation of GIST samples.

Results of the correlation between TFRC-ir and histopathological GIST characteristics are summarized in Table 1. Data on some histopathological criteria were missing for a few GIST samples. While no association was found in the mean size tumor between TFRC-ir, or between non-metastatic/metastatic GISTs, male/female, mutational status, or histological types, we found that the level of TFRC expression was associated with the mitotic index (*p*-value = 0.00061) and risk classification (*p*-value = 0.0228). Analyses of these correlations revealed that TFRC+++ was associated with a high mitotic index (>5/5 mm²), while a lower expression of the TFRC (TFRC+) was associated with a lower mitotic index (≤5/5 mm²) (Figure 10A). Moreover, TFRC+++ was correlated with high-risk cases of GISTs, while TFRC+ was associated with low-risk, very low risk, and no-risk GIST samples (Figure 10B). We next investigated the link between TFRC and YAP expressions or activity in human GIST samples. YAP and TFRC expression (*p*-value < 0.0001), YAP activity, and TFRC expression were correlated (*p*-value = 0.00017) (Table 2). Analyses of contingency tables showed that TFRC+++ was associated with YAP++ staining (Figure 10C). TFRC++ was correlated with YAP++/+ staining, whereas TFRC+ was associated with the absence of YAP expression in GIST samples (Figure 10C). Active YAP (nuclear and mixed location) was associated with a TFRC+++/++, while inactive YAP (cytoplasmic location) was associated with a TFRC+. TFRC++ showed an association with both active and inactive YAP (Figure 10D). To summarize, our data reveal that a high level of TFRC is associated with an elevated mitotic index, high-risk GIST samples, and highly expressed and active YAP. Conversely, a weak expression of the TFRC is associated with a lower mitotic index; a lower-risk GIST; and with a low, inactive, or absent YAP.

## 4. Discussion

### 4.1. Massive Cytotoxicity Induced by the GPX4 Inhibitor, RSL3 in GIST882 and GIST48 Cells

Recent studies support the potential of targeting ferroptosis in multiple cancers, such as triple-negative breast cancer [35], high-risk neuroblastoma [48], rhabdomyosarcoma [36], and others [34,49]. Cancer cells appear to be more sensitive to ferroptosis than normal cells, due to their “addiction to iron” required for proliferation on the one hand and their dependency on antioxidant systems on the other [28,29,50]. Ferroptosis can counteract apoptosis resistance [18,51] and an induction of ferroptosis can sensitize cancer cells to their first-line therapy (radiotherapy and chemotherapy) [49,52,53,54]. It is of note that non-epithelial (hematopoietic and lymphoid, bone tissue, ovary, soft tissue, etc.) cancer cell lines have been shown to be more sensitive to ferroptosis than epithelial carcinomas [55]. Given that primary or secondary resistance to imatinib remains a major concern in GISTs, ferroptosis deserves further consideration in this type of cancer. A recent study from Ishida et al. [26] demonstrated that the GPX4 inhibitor RSL3 induced ferroptotic cell death in imatinib-sensitive GIST-T1 cells and imatinib-resistant GIST-R8. Lipid peroxidation was not reported in that study. Their observations are in line with our data which demonstrate that RSL3 induced ferroptosis through important lipid peroxidation in the imatinib-sensitive and -resistant human GIST cells, GIST882 cells, and GIST48 cells, respectively. Moreover, we showed that Lip, an antioxidant, reversed cytotoxicity and lipid peroxidation induced by RSL3. RSL3 treatment appeared to be efficient to inhibit tumor growth in BjeLR cell xenografts in mice [56], in prostate tumor models in vivo [57], in glioblastoma [58], and in GIST-T1 xenografts after imatinib therapy cessation [26]. Our in vitro data support the view that GPX4 inhibitors should be further evaluated in GIST xenograft models in vivo.

### 4.2. Contribution of Ferroptosis to a Reduction in Cell Viability Mediated by VP and CA3 in GIST882 and GIST48 Cells

We previously reported that the YAP inhibitor VP induced massive cell death in both GIST882 and GIST48 cells, but the type of cell death involved remained unknown [9]. Here, we established that VP cytotoxicity is mediated by ferroptosis and confirmed that VP behaves like a canonical YAP inhibitor in GIST882 and GIST48 cells, since VP decreased the expression level of several known YAP transcriptional targets, as reported in other cell lines [41,59].

CA3, recently reported as a YAP inhibitor [46,60,61,62], also demonstrated strong lipid peroxidation and marked a reduced viability reversed by ferroptosis inhibitors in both GIST882 and GIST48 cell lines. Surprisingly, in view of the current literature, we observed a paradoxical increase, instead of a decrease, in the YAP transcriptional targets CTGF and CYR61, as well as in AMOTL2 mRNA expressions after CA3 treatment in GIST882, while no change was detected in GIST48. These results highlight that ferroptosis induced by CA3 appears to be uncoupled from the inhibition of the YAP transcriptional program in GIST882 and GIST48 cells. Further studies will be required to unravel the CA3 mechanism of action in these cell lines.

A positive regulation of YAP on ferroptosis has been reported in basal and luminal breast cancer, non-small cell lung cancer, clear-cell renal carcinoma [16] and in mesothelioma cells [63]. Conversely, Gao et al. [64] identified YAP as a negative ferroptosis regulator in hepatocellular carcinoma cells, in line with our present original data VP in GISTs.

Our data show that VP significantly increased ferrous iron levels in both GIST cell lines, which is in line with recent studies showing that inhibition of YAP sensitized cells to ferroptosis by increasing the labile iron pool [65,66].

VP treatment also decreased TFRC mRNA expression, which is probably a defense mechanism to reduce iron uptake. However, the protective effect on cell viability elicited by the iron chelator DFO was not complete, suggesting that, although ferroptosis largely contributes to VP-mediated cytotoxicity, other cell death mechanisms might also be involved. Apoptosis was ruled out in GIST48 cells, but other cell death mechanisms remain to be explored, as YAP has been shown to be associated with multiple cell death mechanisms [67].

### 4.3. Lipid Peroxidation and GSH Depletion, Two Hallmarks of Ferroptosis, Observed in VP- and CA3-Treated Cells

Flow cytometry assays showed an increase in lipid peroxidation in GIST882 and GIST48 cells after both VP and CA3 treatments. CA3-mediated lipid peroxidation was completely inhibited by Lip and DFO, suggesting that ferroptosis is the major cell death mechanism induced by CA3 in GIST cells. However, those ferroptosis inhibitors were unable to completely inhibit lipid peroxidation in GIST882 and GIST48 cells after VP treatment. The larger increase in labile iron pool in VP-treated cells compared to CA3-treated cells could be responsible for a larger ROS production through the Fenton reaction and an increased activity of iron-containing lipid peroxidases, which are crucial for lipid peroxidation induction [68]. We hypothesized that this would prevent DFO and Lip to fully counteract lipid peroxidation. Moreover, we showed that the GSH content was reduced by CA3 or VP treatment in GIST882 and GIST48 cells. Our in vitro data are in line with the results of Morishita et al. [45], who reported that a depletion in GSH was required for VP cytotoxicity in acute lymphoblastic leukemia patient-derived xenografts.

### 4.4. The Depletion in GPX4, but Not SLC7A11, and Protein Expression in Response to CA3 and VP

A major antioxidant pathway that prevents ferroptosis is the SLC7A11/GSH/GPX4 axis [20]. In this study, no modification in SLC7A11 mRNA or protein expression was detected, despite a significant decrease in the GSH content. This suggests that VP and CA3 could modulate other genes involved in GSH biosynthesis. Conversely, a significant depletion in GPX4 protein expression was observed in both GIST cell lines after treatment with VP or CA3, while GPX4 mRNA expression remained unaffected. These results are consistent with the degradation of the GPX4 protein level, independently of GPX4 mRNA changes, already observed in fibrosarcoma cells and BJeLR-engineered transformed fibroblast cells after treatment with another class II ferroptosis inducer, FIN-56 [69]. It is noteworthy that FIN-56 is an analog of “caspase-3/7-independent lethals” compound CIL-56, otherwise known as CA3, the drug used in our study. Although the link between CA3 (CIL-56) and ferroptosis has been controversial [70,71], our study provides evidence that CA3 (CIL-56) induces ferroptosis with a significant depletion in GPX4 protein levels in GIST cells. The mechanisms underlying GPX4 protein reduction after treatment with VP or CA3 in GIST cell lines remain to be clarified. It has been reported in other models that GPX4 can be degraded via the ubiquitin–proteasome system, as shown in triple-negative breast cancer [72,73]. Moreover, some studies described mTORC1 as a key player in GPX4 protein synthesis [74] and in the prevention of autophagy-mediated GPX4 degradation [73,75].

### 4.5. Proposed Model Linking VP or CA3 Treatment to Ferroptosis in GIST Cells

**Figure 11:**
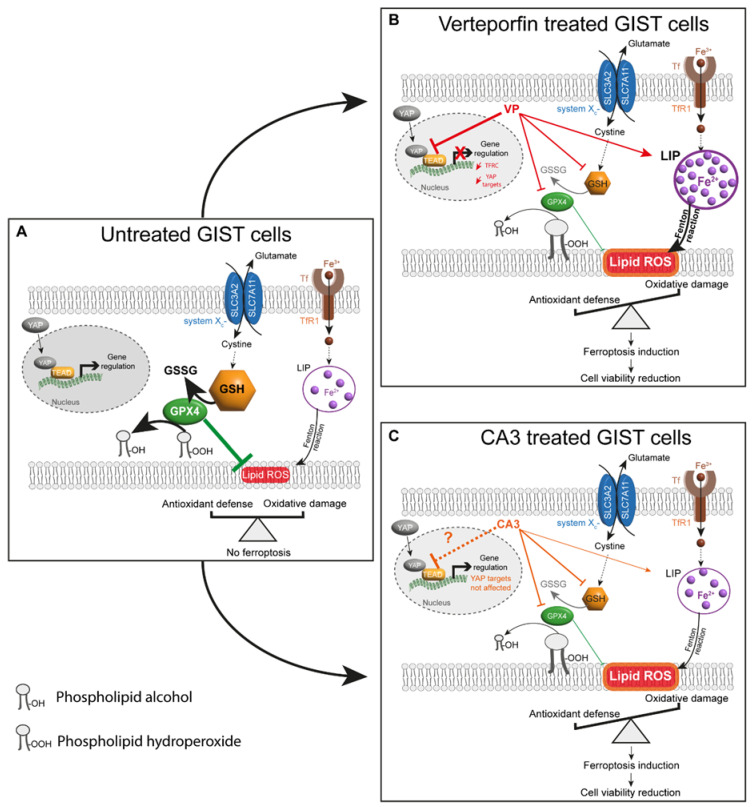
Proposed model for ferroptosis induction by VP or CA3 in GIST human cell lines. (**A**) In untreated GIST cells, a balance between oxidative damage through lipid ROS/lipid peroxidation and antioxidant defenses, including the SLC7A11/GSH/GPX4 axis, prevents ferroptosis. Furthermore, YAP can interact with TEAD and regulate gene expression. (**B**) GIST882 and GIST48 treatment with VP induced an increase in LIP, composed mainly of ferrous iron, and a significant reduction in the GSH content and GPX4 protein level, which are responsible for increasing the lipid peroxidation. As a result of an impaired balance between oxidative damage and antioxidant defenses, ferroptosis induction mediated a reduction in cell viability. The effect of VP on ferroptosis might be due to YAP inhibition, as YAP target genes were downregulated. Additionally, TFRC gene expression was reduced, probably in response to the elevated free ferrous iron. (**C**) In CA3-treated cells, the free ferrous iron concentration difference was not statistically significant in GIST48, and no modification was observed in GIST882. However, the GSH content and GPX4 protein level were significantly reduced, which may be responsible for an increase in the lipid peroxidation. As a result of an impaired balance between oxidative damage and antioxidant defenses, ferroptosis induction mediated a cell viability reduction. However, in GIST882 and GIST48 cells, the CA3 effect on ferroptosis induction might be independent of an inhibition of the YAP transcriptional program. Thick arrows indicate a stronger effect of the drug. Thin arrows indicate a lesser effect of the drug. Red and orange arrows indicate the different effects mediated by VP and CA3, respectively. The dashed black arrow indicates that intermediate steps are hidden. The dashed orange arrow indicates that, in contrast to the literature, our original results show that CA3 does not inhibit the YAP transcriptional program in GIST882 and GIST48 cell lines. GSH: glutathione; GSSG: glutathione disulfide; LIP: labile iron pool; Tf: transferrin; TFRC: transferrin receptor 1; Fe^2+^: ferrous iron; Fe^3+^: ferric iron.

A model which emerged from our results demonstrated a link between the inhibition of the YAP pathway and ferroptosis in GIST cells after VP treatment (Figure 11B). The inhibition of YAP transcriptional program mediated by VP reduced GIST cell viability and increased lipid peroxidation, which were partially reversed by ferroptosis inhibitors. Moreover, VP-treated GIST cells were characterized by an increase in free ferrous iron concentration and a depletion in the GSH content and GPX4 protein levels. We also highlight a link between CA3 and ferroptosis induction through a significant increase in lipid peroxidation, a slight increase in the labile iron pool, and a major depletion in the GSH content and GPX4 protein levels, independently of the inhibition of the transcriptional program downstream of YAP. These results suggest that both VP and CA3 impair the balance between iron-mediated oxidative damage and antioxidant defenses through different molecular mechanisms in GIST882 and GIST48 cells, leading to further lipid peroxidation and ferroptosis as a cell death mechanism.

### 4.6. TFRC Expression in Human GIST Tissue and Its Positive Correlation with Mitotic Counts, Risk Classification, and YAP Expression and Activation

Iron is essential for biological processes including DNA synthesis and DNA repair, which are important for cell growth and proliferation. Due to the high rate of proliferation in cancer cells, these cells appear to be “addicted to iron” [28,50]. Elevated iron in cancer cells allows tumor growth and progression. In cancer cells, iron is mainly imported via the TFRC, and this transmembrane receptor has been found to be overexpressed in many types of cancers, such as brain, breast, colon, ovarian, and lung cancers, as well as in leukemia [47]. For those reasons, and also due to its extracellular accessibility and ability to internalize, the TFRC has been used as a therapeutic target in cancers [76]. To the best of our knowledge, TFRC protein expression has never been explored in human GIST tissues. Our IHC results show that the TFRC was expressed in all human GIST tissues included in our cohort. We demonstrate that a high level of TFRC is associated with high-risk GISTs, while a low level of TFRC is associated with very low risk, low-risk, and no-risk GIST samples. This is in line with what was observed in other types of cancers, such as breast, liver, and serous ovarian cancers, as well as esophageal squamous cell carcinoma [29,47,77]. In addition, a positive association was found between a high level of TFRC and an elevated mitotic index, which is a major element in the assessment of the risk of relapse in GISTs [1,37,78]. Overall, we can speculate that a high level of TFRC is associated with poor prognosis in GISTs. Moreover, our studies on GISTs reveal a positive correlation between TFRC expression and YAP expression and activation, which was not explored in other malignancies. This appears consistent with the literature since the TFRC is known to be a target of YAP [63,79].

## 5. Conclusions

In conclusion, our in vitro data establish a role of ferroptosis induction in GISTs. Given the potential of targeting ferroptosis in various types of cancers [20], it is tempting to speculate that, also in GISTs, ferroptosis could be considered as a mechanism subjected to therapeutic targeting. We provide evidence that the pharmacological inhibition of YAP by VP induces ferroptosis in two human GIST cell lines, while CA3 induces ferroptosis independently of YAP inhibition. Moreover, the study of TFRC expression in a human cohort of tissue samples from primary and metastatic GIST tumors reveals that the TFRC could be an attractive therapeutic target in high-risk GISTs and in GISTs with an elevated mitotic index. Finally, our study on GIST TMA shows that a high TFRC expression was positively correlated with YAP nuclear localization, reflecting YAP activation.

## Figures and Tables

**Table 1:** The association of TFRC-ir with clinicopathologic characteristics in GIST TMA samples (Bordet + KU Leuven).

	TFRC +++	TFRC ++	TFRC +	N.A. (*n*)	*p*-Value *
**Mean tumor size (mm)**	105.83	48.25	79.8	17	0.268
**Site of origin (TMA sample)**	TFRC +++	TFRC ++	TFRC +	Total	*p*-Value ^†^
	*n* (%)	*n* (%)	*n* (%)	*n* (%)	
Non-metastatic	12 (14%)	30 (36%)	23 (27%)	65 (77%)	
Metastatic	0 (0%)	13 (15%)	7 (8%)	20 (23%)	
Total	12 (14%)	43 (51%)	30 (35%)	85 (100%)	0.09
**Sex**	TFRC +++	TFRC ++	TFRC +	Total	*p*-Value ^†^
	*n* (%)	*n* (%)	*n* (%)	*n* (%)	
Male	7 (8%)	25 (30%)	10 (12%)	42 (50%)	
Female	5 (6%)	17 (20%)	20 (24%)	42 (50%)	
Total	12 (14%)	42 (50%)	30 (36%)	84 (100%)	0.074
**Mitotic index**	TFRC +++	TFRC ++	TFRC +	Total	*p*-Value ^†^
	*n* (%)	*n* (%)	*n* (%)	*n* (%)	
≤5/5 mm²	5 (7%)	24 (33%)	24 (33%)	45 (73%)	
>5/5 mm²	7 (10%)	11 (16%)	1 (1%)	20 (27%)	
Total	12 (17%)	35 (49%)	25 (34%)	65 (100%)	0.00061
**Histological type**	TFRC +++	TFRC ++	TFRC +	Total	*p*-Value ^†^
	*n* (%)	*n* (%)	*n* (%)	*n* (%)	
Epitheloid	2 (2%)	2 (2%)	1 (1%)	5 (5%)	
Spindle	6 (7%)	36 (44%)	26 (32%)	68 (83%)	
Mixed	3 (4%)	3 (4%)	3 (4%)	9 (12%)	
Total	11 (13%)	41 (50%)	30 (37%)	82	0.104
**Risk classification (Miettinen [36])**	TFRC +++	TFRC ++	TFRC +	Total	*p*-Value ^†^
	*n* (%)	*n* (%)	*n* (%)	*n* (%)	
High risk	8 (13%)	7 (12%)	3 (5%)	18 (30%)	
Moderate risk	0 (0%)	7 (12%)	4 (7%)	11 (19%)	
Low, very low, or no risk	4 (7%)	13 (21%)	14 (23%)	31 (51%)	
Total	12 (20%)	27 (45%)	21 (35%)	60 (100%)	0.0228
**Mutation status**	TFRC +++	TFRC ++	TFRC +	Total	*p*-Value ^†^
	*n* (%)	*n* (%)	*n* (%)	*n* (%)	
KIT mutation	3 (4%)	24 (36%)	22 (33%)	49 (73%)	
PDGFR mutation	0 (0%)	5 (7%)	6 (9%)	11 (16%)	
No *KIT/PDGFRA* mutation	1 (1%)	3 (5%)	3 (5%)	7 (11%)	
Total	4 (5%)	32 (48%)	31 (47%)	67 (100%)	0.786

* Kruskal–Wallis test; † Freeman–Halton extension of Fisher’s test.

**Table 2:** The association of TFRC-ir with YAP-ir, KIT-ir, and YAP activation in 87 GIST cases.

YAP-ir	TFRC +++	TFRC ++	TFRC +	Total	*p*-Value ^†^
	***n* (%)**	***n* (%)**	***n* (%)**	***n* (%)**	
YAP ++	7 (8%)	7 (8%)	0 (0%)	14 (16%)	
YAP +	5 (6%)	35 (40%)	23 (27%)	63 (73%)	
YAP −	1 (1%)	1 (1%)	8 (9%)	10 (11%)	
Total	13 (14%)	43 (50%)	31 (36%)	87 (100%)	<0.0001
**YAP activation**	**TFRC +++**	**TFRC ++**	**TFRC +**	**Total**	***p*-Value ^†^**
	***n* (%)**	***n* (%)**	***n* (%)**	***n* (%)**	
Active YAP (M + N)	11 (14%)	15 (20%)	5 (7%)	31 (41%)	
Inactive YAP (D)	1 (1%)	27 (35%)	18 (23%)	46 (59%)	
Total	12 (15%)	42 (55%)	23 (30%)	77 (100%)	0.00017
**KIT-ir**	**TFRC +++**	**TFRC ++**	**TFRC +**	**Total**	***p*-Value ^†^**
	***n* (%)**	***n* (%)**	***n* (%)**	***n* (%)**	
KIT ++	7 (7%)	26 (31%)	11 (13%)	44 (51%)	
KIT +	6 (7%)	17 (19%)	20 (23%)	43 (49%)	
KIT −	0 (0%)	0 (0%)	0 (0%)	0 (0%)	
Total	13 (14%)	43 (50%)	31 (36%)	87 (100%)	0.104

† Freeman–Halton extension of Fisher’s test.

## Data Availability

The data used to support the findings of this study are included within this article and supplementary files.

## Supplementary Materials

The following supporting information can be downloaded at: https://www.mdpi.com/article/10.3390/cancers14205050/s1. Figure S1: C11-BODIPY analysis for GIST882 cells treated with CumOOH, a strong oxidant, used as a positive control for lipid peroxidation. Figure S2: C11-BODIPY analysis for GIST48 cells treated with CumOOH, a strong oxidant, used as a positive control for lipid peroxidation. Figure S3: VP and CA3 did not induce apoptosis in GIST48 cells. Figure S4: RNA sequencing analysis in GIST882 cells. Figure S5: RNA sequencing analysis in GIST48 cells. Figure S6: YAP targets genes mRNA relative expression after VP or CA3 treatment in GIST882 and GIST48 cells. Table S1: Clinicopathological features of the Bordet Institute’s FFPE GIST slides. Table S2: Clinicopathologic features of Bordet GIST TMA (20 GIST cases). Table S3: Clinicopathologic features of KU Leuven GIST TMA (76 GIST cases). Table S4: Primary and secondary antibodies used for IHC. Table S5: Primers used for qPCR. Table S6: Primary and secondary antibodies used for WB. All the whole western blot figures can be found in the supplementary materials.
