# Supplementary material for: Ferroptosis Induction and YAP Inhibition as New Therapeutic Targets in Gastrointestinal Stromal Tumors (GISTs)
Source: Cancers (Basel). 2022 Oct 15;14(20):5050. doi: 10.3390/cancers14205050 (PMC9599726; doi:10.3390/cancers14205050)
Supplement: Supplementary file 1 [file cancers-14-05050-s001.zip › Manuscript 1916180_Supplementary Tables and Figures_ revised 221005.pdf]

cancers

MDPI

Supplementary Figures

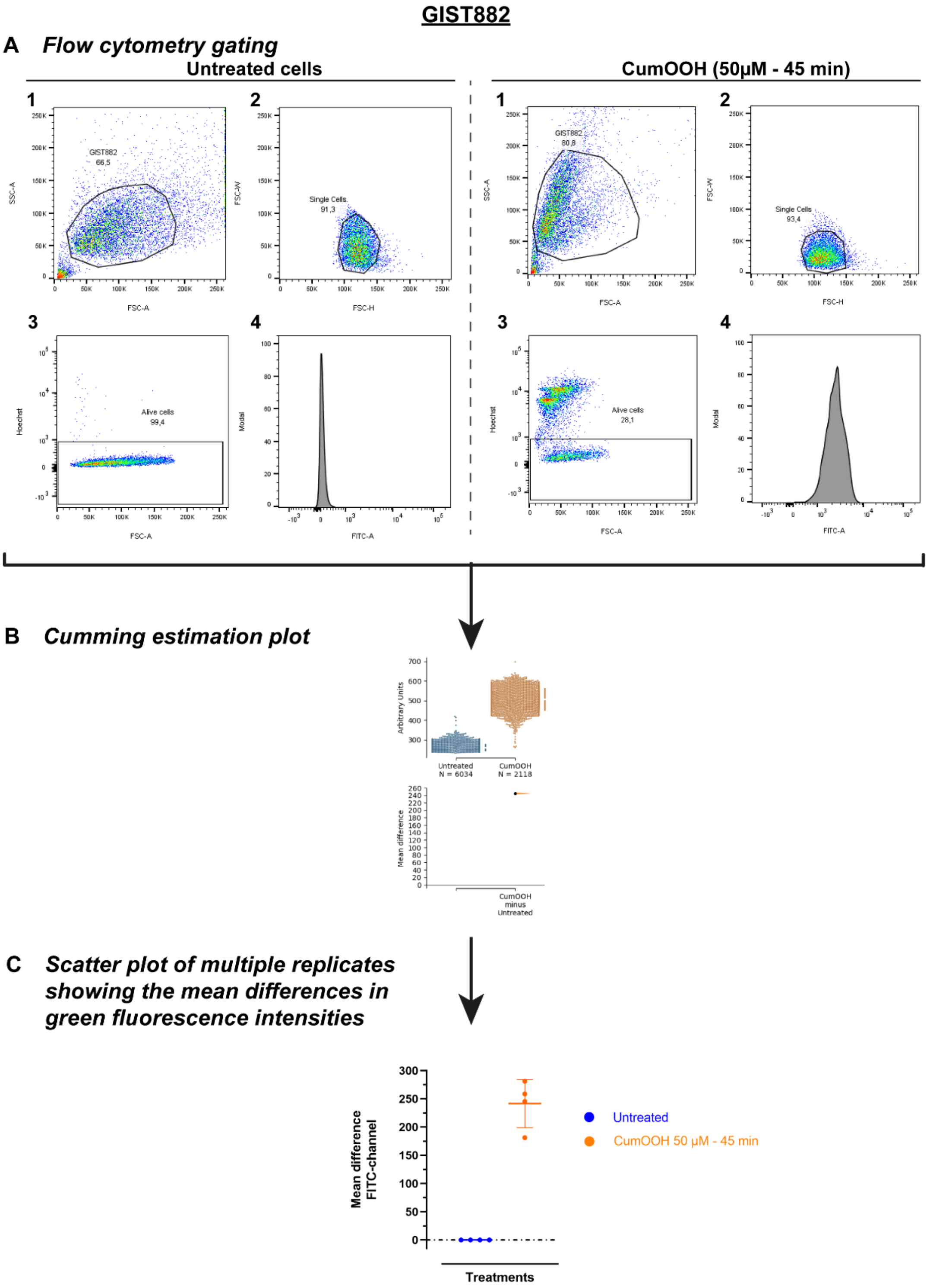

Figure S1. C11-BODIPY analysis for GIST882 cells treated with CumOOH, a strong oxidant, used as positive control for lipid peroxidation (A) Flow cytometry gating. Left panel: Untreated GIST882. Right panel: GIST882 cells treated with CumOOH (50 µM) for 45 min. Each panel described four steps of gating for C11-BODIPY flow cytometry analysis. First step (1) allowed the selection of the cell population of interest and excluded debris based on a SSC-A vs FSC-A plot. The second step (2) excluded doublet cells based on a FSC-W vs FSC-H plot. Living cells were selected based on Hoechst exclusion in the third step (3) and were showed in a boxed gate on a Hoechst vs FSC-A plot. Finally,

single and living GIST882 with a green fluorescence intensity are shown in step 4 as a histogram for FITC-A channel. (B) Data from step 4 are used to generate Cumming estimation plots with DABEST. On the upper axes, all datapoints are presented as a swarm plot and displayed the underlying distribution. The effect size (mean difference) is presented as a bootstrap sampling distribution. The number of events (single cells) analyzed is indicated in the Figure. Mean difference between CumOOH treated GIST882 and untreated GIST882 is depicted as a dot with 95% confidence interval (95% CI) indicated by the ends of the vertical error bars on the lower axis. (C) Mean differences in green fluorescence intensity of multiple independent experiments are combined as shown in a scatter plot and presented as mean values ± SD.

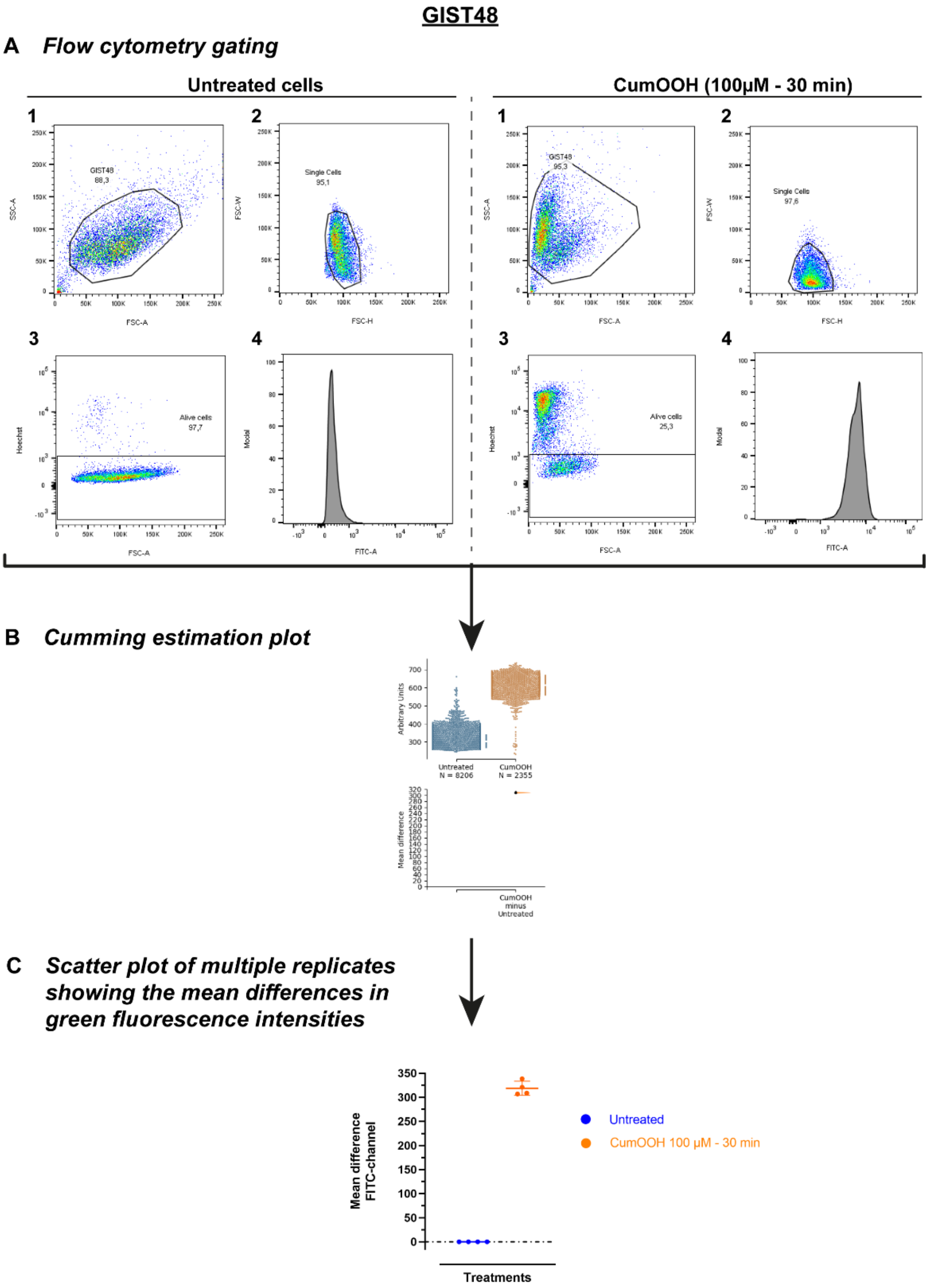

Figure S2. C11-BODIPY analysis for GIST48 cells treated with CumOOH, a strong oxidant, used as a positive control for lipid peroxidation (A) Flow cytometry gating. Left panel: Untreated GIST48. Right panel: GIST48 cells treated with CumOOH (100 µM) for 30 min. Each panel described four

steps of gating for C11-BODIPY flow cytometry analysis. First step (1) allowed the selection of the cell population of interest and excluded debris based on a SSC-A vs FSC-A plot. The second step (2) excluded doublet cells based on FSC-W vs FSC-H plot. Living cells were selected based on Hoechst exclusion in the third step (3) and are shown in a boxed gate on a Hoechst vs FSC-A plot. Finally, single and living GIST48 with a green fluorescence intensity are shown in step 4 as a histogram for FITC-A channel. (B) Data from step 4 are used to generate Cumming estimation plots with DABEST. On the upper axes, all datapoints are presented as a swarm plot and displayed the underlying distribution. The effect size (mean difference) is presented as a bootstrap sampling distribution. The number of events (single cells) analyzed is indicated in the Figure. Mean difference between CumOOH treated GIST48 and untreated GIST48 is depicted as a dot with 95% confidence interval (95% CI) indicated by the ends of the vertical error bars on the lower axis. (C) Mean differences in green fluorescence intensity of multiple independent experiments are combined as shown in a scatter plot and presented as mean values ± SD.

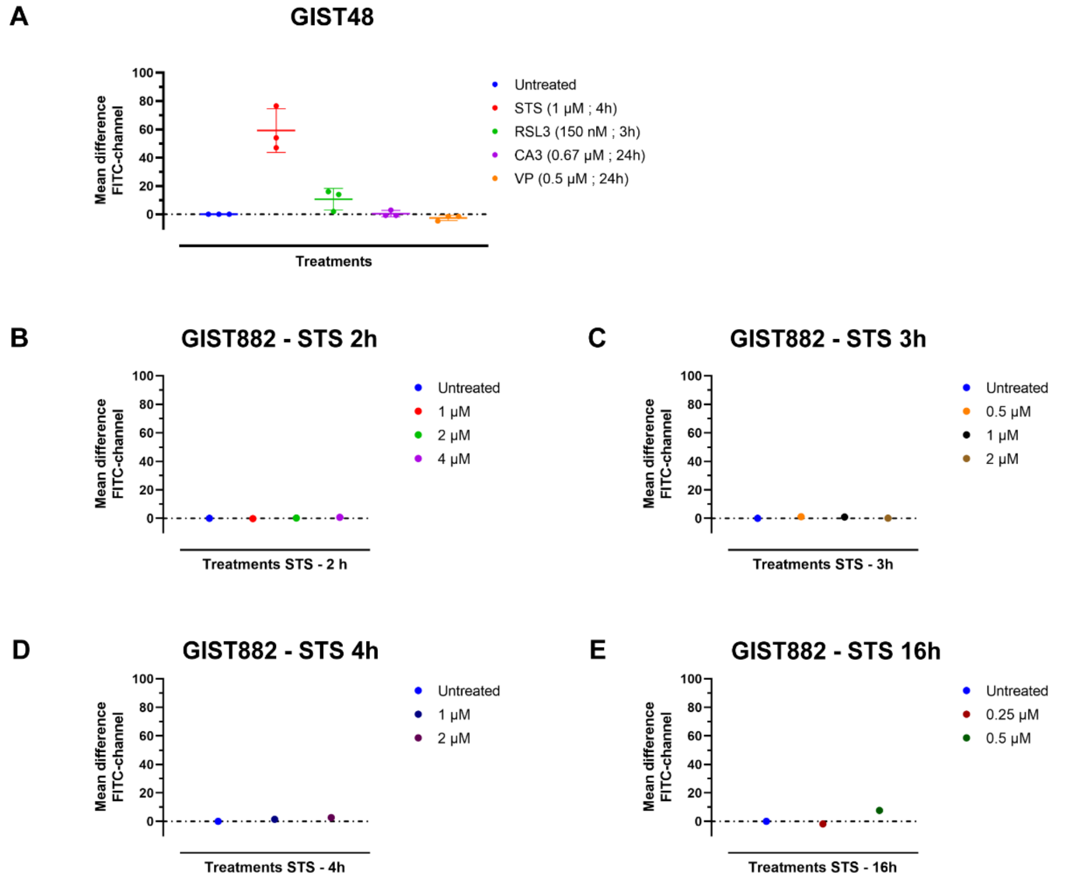

Figure S3. VP and CA3 did not induced apoptosis in GIST48 cells. Active caspase-3 apoptosis staining analyzed by flow cytometry. (A) GIST48 cells were treated with STS 1 µM for 4 h, RSL3 150 nM for 3 h, CA3 0.67 µM for 24 h and VP 0.5 µM for 24 h. Data were analyzed with DABEST software and scatter plots were generated using Prism 9 software. Data are presented as mean values ± SD. Mean values from three independent experiments. (B-E) GIST882 were treated with different indicated concentrations of STS during 2 h (B), 3 h (C), 4 h (D) and 16 h(E). Data were analyzed with DABEST software and scatter plots were generated using Prism 9 software. Mean difference in green fluorescence is presented.

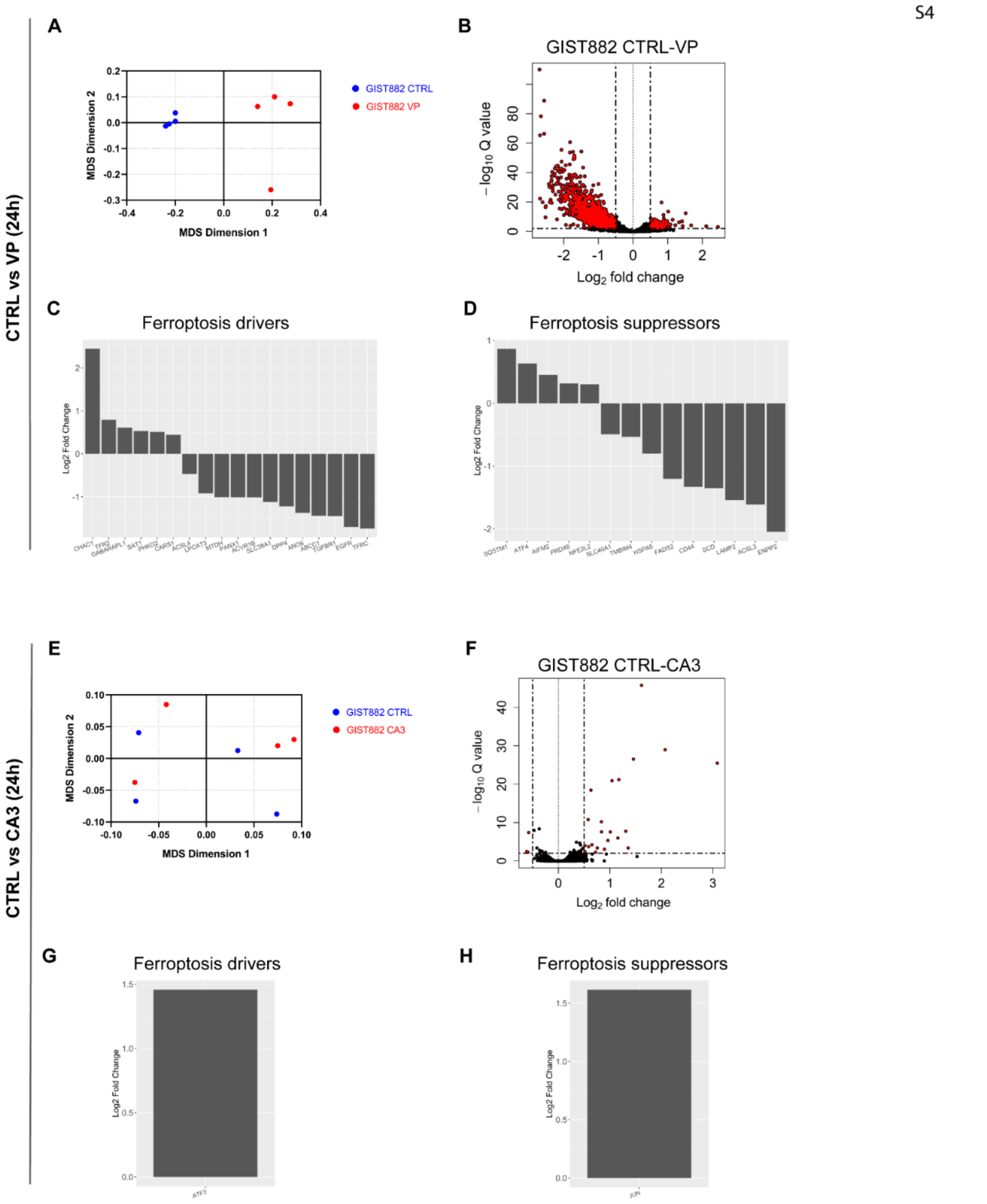

Figure S4. RNA sequencing analysis in GIST882 cells. Result of RNA sequencing in GIST882 treated with VP 2 μM for 24 hours (upper panels) or with CA3 670 nM for 24 hours (lower panels). (A,E) Multidimensional scaling plots (MDS). (B,F) Volcano plots representing the differential expressed genes (DEG). Significant DEG (q-value ≤ 0.01 and log2 FC > 0.5) are indicated with red dots. Black dots represent non-significant DEG. Black dot lines indicate the threshold of significance. (C,D) Ferroptosis drivers and suppressors differentially expressed between control and VP-treated GIST882 cells with the corresponding log2 FC. (G,H) Ferroptosis drivers and suppressors differentially expressed between control and CA3-treated GIST882 cells with the corresponding log2 FC.

S4

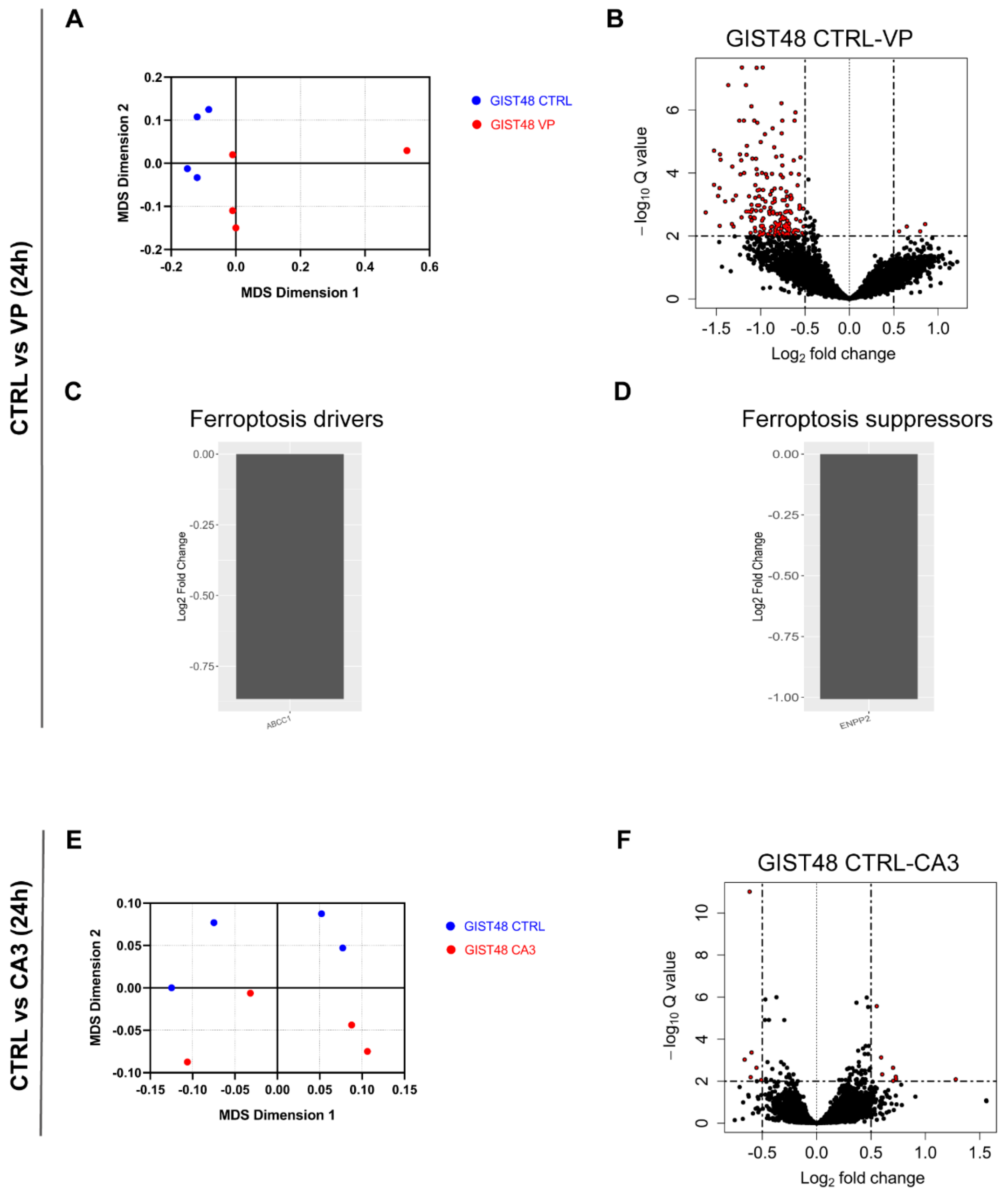

Figure S5. RNA sequencing analysis in GIST48 cells. Result of RNA sequencing in GIST48 treated with VP 0.5 µM for 24 hours (upper panels) or with CA3 670 nM for 24 hours (lower panels). (A,E) Multidimensional scaling plots (MDS). (B,F) Volcano plots representing the differential expressed genes (DEG). Significant DEG (q-value ≤ 0.01 and log2 FC > 0.5) are indicated with red dots. Black dots represent non-significant DEG. Black dot lines indicate the threshold of significance. (C,D) Ferroptosis drivers and suppressors differentially expressed between control and VP-treated GIST48 cells with the corresponding log2 FC. No significant DEG related to ferroptosis was found between control and CA3-treated GIST48 cells.

Figure S6. YAP targets genes mRNA relative expression after VP or CA3 treatment in GIST882 and GIST48 cells. GIST882 and GIST48 cells were treated with VP (2 µM or 0.5 µM for GIST882 and

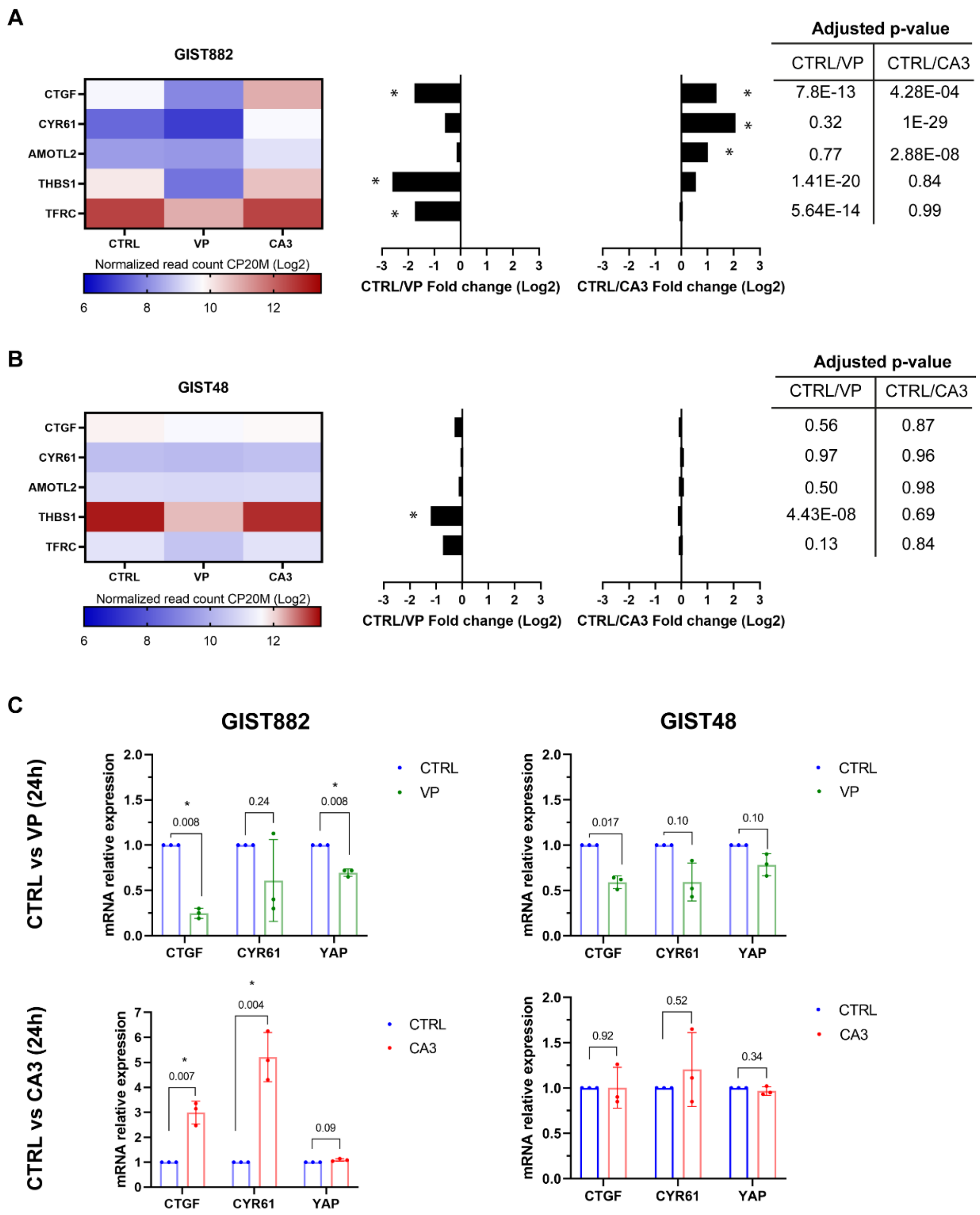

GIST48, respectively) or CA3 (670 nM for GIST882 and GIST48) for 24 h. (A) mRNAs relative expression of indicated YAP targets genes were analyzed by RNA sequencing in GIST882 cells. Left panel represents heatmap of normalized read count of each gene and is represented in a Log2 scale. Middle panels are mRNAs relative expression of YAP targets genes after VP and CA3 treatment relative to control GIST882 cells. Right panel shows the adjusted p-value for each gene after VP and CA3 treatment. Data presented as mean fold change are shown from four biological replicates. Asterisks indicate significant differential expressed genes with a fold change > 0.5 and an adjusted p-value < 0.01. (B) mRNAs relative expression of indicated YAP targets genes were analyzed by RNA sequencing in GIST48 cells. Left panel represents heatmap of normalized read count of each gene and is represented in a Log2 scale. Middle panels are mRNAs relative expression of YAP targets genes after VP and CA3 treatment relative to control GIST48 cells. Right panel shows the adjusted p-value for each gene after VP and CA3 treatment. Data presented as mean fold changes are shown from four biological replicates. Asterisks indicate significant differential expressed genes with a fold change > 0.5 and an adjusted p-value < 0.01. (C) mRNAs relative expression of indicated YAP targets genes were analyzed by qPCR and presented as fold change (treatment to control cells). p values were calculated using multiple ratio paired t-test. p-values < 0.01 considered statistically significant.

Data presented as mean values ± SD are shown from four or five independent experiments with technical triplicates for each experiment.

Supplementary Tables

Table S1. Clinicopathological features of Bordet Institute's FFPE GIST slides

Case number	Age	Sex	Location	Morphology	Diagnostic	Type	Mitotic Index
**1**	71	F	Stomach	Spindle	Low risk	Primary	No mitosis
**2**	59	F	Stomach	Spindle	Low risk	Primary	1/50 fields
**3**	57	F	Small intestine	Spindle	High risk	Primary	>5/5mm^2
**4**	68	F	Stomach	Spindle	High risk	Primary	>5/5mm^2
**5**	71	M	Colon	Spindle	High risk	Primary	>5/5mm^2

Table S2. Clinicopathologic features of Bordet GIST TMA (20 GIST cases)

**Bordet GIST TMA**	
**Mean tumour size (mm^2)**	76.5
N.A mean tumour size (n)	0
**Sex/Age average**	Total (n)/years
Male	11/63.9
Female	9/64.5
N.A.	0
**Site of origin (TMA sample)**	Total (n)
Gastric	10
Small Bowel	7
Colon	1
Rectal	1
Oesophagus	0
Extragastrointestinal	0
Metastasis or recurrence	1
N.A.	0
**Tumor morphology**	Total (n)
Spindle	15
Epithelioid	2
Mixed	3
N.A.	0
**Risk category (Miettinen [36])**	Total (n)
Very low - No risk	2
Low risk	7
Moderate risk	3
High risk	8
N.A.	0
**Mitotic index**	Total (n)
≤5/5mm2	11
>5/5mm2	9
N.A.	0
**Mutation status**	Total (n)

KIT mutation	N.A.
PDGFRA mutation	N.A.
No *KIT/PDGFRA* mutation	N.A.
N.A.	20

Table S3. Clinicopathologic features of KU Leuven GIST TMA (76 GIST cases)

**KU Leuven GIST TMA**	
**Mean tumour size (mm²)**	68.5
N.A mean tumour size (n)	17
**Sex/Age average**	Total (n)/years
Male	38/61
Female	35/62
N.A.	3
**Site of origin**	Total (n)
Gastric	30
Small Bowel	9
Colon	0
Rectal	2
Oesophagus	1
Extragastrointestinal	5
Metastasis or recurrence	27
N.A.	2
**Tumor morphology**	Total (n)
Spindle	61
Epithelioid	3
Mixed	6
N.A.	6
**Risk category (Miettinen [36])**	Total (n)
Very low - No risk	11
Low risk	12
Moderate risk	8
High risk	10
N.A.	8
**Mitotic index**	Total (n)
≤5/5mm2	43
>5/5mm2	14
N.A.	19
**Mutation status**	Total (n)
KIT mutation	57
PDGFRA mutation	12
No *KIT/PDGFRA* mutation	7
N.A.	0

Table S4. Primary and secondary antibodies used for IHC

Primary antibodies	Supplier	Cat. N°	Host	Dilution
**KIT**	DAKO	A4502	Rabbit	1/500
**Anti-Transferrin Receptor antibody**	Abcam	ab84036	Rabbit	1/250
**Recombinant Anti-YAP1 antibody [EP1674Y]**	Abcam	ab52771	Rabbit	1/500
**Recombinant Anti-Nrf2 antibody [EP1808Y] - ChIP Grade**	Abcam	ab62352	Mouse	1/125
**Secondary antibodies**	Supplier	Cat. N°	Host	Dilution
**Anti-rabbit Biotin-SP**	Jackson Immunoresearch laboratories, Inc.	711-065-152	Donkey	1/200
**Anti-mouse Biotin-SP**	Jackson Immunoresearch laboratories, Inc.	715-065-150	Donkey	1/200

Table S5. Primers used for qPCR

Primers	Sequences
**h_HPRT1_fwd**	CATTATGCTGAGGATTTGGAAAGG
**h_HPRT1_rev**	CTTGAGCACACAGAGGGCTACA
**h_GAPDH_fwd**	ACCCACTCCTCCACCTTTGAC
**h_GAPDH_rev**	CATACCAGGAAATGAGCTTGACAA
**h_TFRC_fwd**	ACCATTGTCATATACCCGGTTCA
**h_TFRC_rev**	CAATAGCCCAAGTAGCCAATCAT
**h_GPX4_fwd**	TAGAAATAGTGGGGCAGGTCC
**h_GPX4_rev**	CGTCAAATTCGATATGTTCAGC
**h_SLC7A11_fwd**	GCAACAAAGATCGGAACTGCT
**h_SLC7A11_rev**	GCTGGCTGGTTTTACCTCAAC
**h_NRF2_fwd**	TCTGACTCCGGCATTTCACT
**h_NRF2_rev**	GGCACTGTCTAGCTCTTCCA
**h_ACSL4_fwd**	GCTATCTCCTCAGACACACCGA
**h_ACSL4_rev**	AGGTGCTCCAACTCTGCCAGTA
**h_HMOX1_fwd**	GGCCAGCAACAAAGTGCAAG
**h_HMOX1_rev**	TGGCATAAAGCCCTACAGCA
**h_FTH1_fwd**	CTTTGACCGCGATGATGTGGCTTT
**h_FTH1_rev**	TTTGTCAGTGGCCAGTTTGTGCAG
**h_ALOX15_fwd**	GGAGCCTTCCTAACCTACAGC
**h_ALOX15_rev**	CTCACGATTCCTTCCACATACC
**h_CTGF_fwd**	TGGAAGAGAACATTAAGAAGGGCA
**h_CTGF_rev**	TGCAGCCAGAAAGCTCAAAC
**h_YAP1_fwd**	TGACCCTCGTTTTGCCATGA
**h_YAP1_rev**	GTTGCTGCTGGTTGGAGTTG

**h_CYR61_fwd**	GCAAGGAGCTGGGATTCGAT
**h_CYR61_rev**	ATTCCAAAAACAGGGAGCCG

Table S6. Primary and secondary antibodies used for WB

Primary antibodies	Supplier	Cat. N°	Host	Dilution
**GAPDH (14C10)**	Cell Signaling Technology	2118	Rabbit	1/500
**Recombinant Anti-Glutathione Peroxidase 4 antibody [EPNCIR144]**	Abcam	ab125066	Rabbit	1/1000
**Anti-xCT antibody (SLC7A11)**	Abcam	ab37185	Rabbit	1/1000
**Secondary antibodies**	Supplier	Cat. N°	Host	Dilution
**Anti-rabbit 680**	Invitrogen	35568	Goat	1/10000
