# Supplementary figures and images for: Ferroptosis Induction and YAP Inhibition as New Therapeutic Targets in Gastrointestinal Stromal Tumors (GISTs)
Source: Cancers (Basel). 2022 Oct 15;14(20):5050. doi: 10.3390/cancers14205050 (PMC9599726; doi:10.3390/cancers14205050)

Western Blots : GPX4

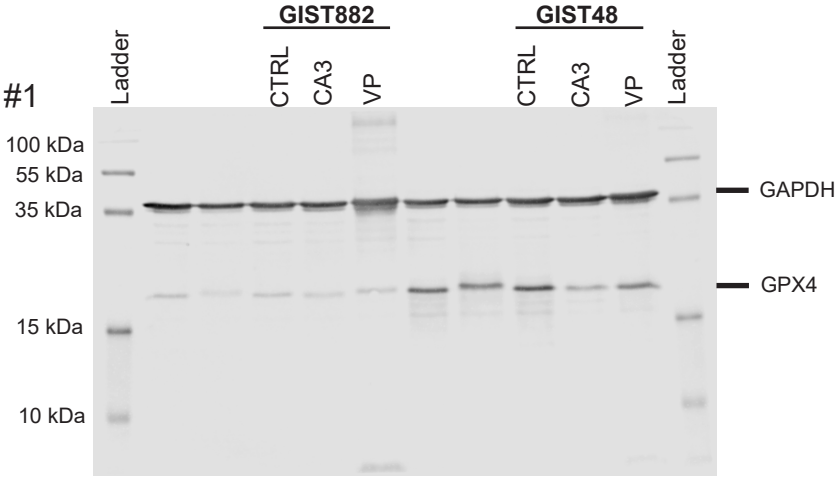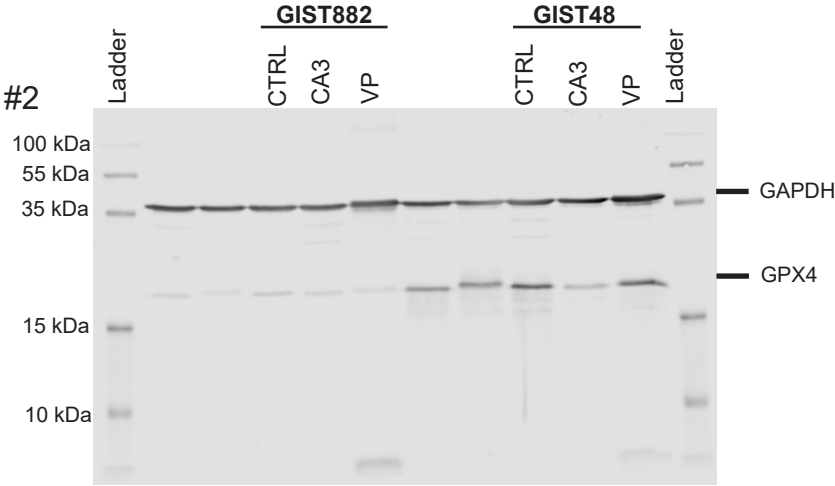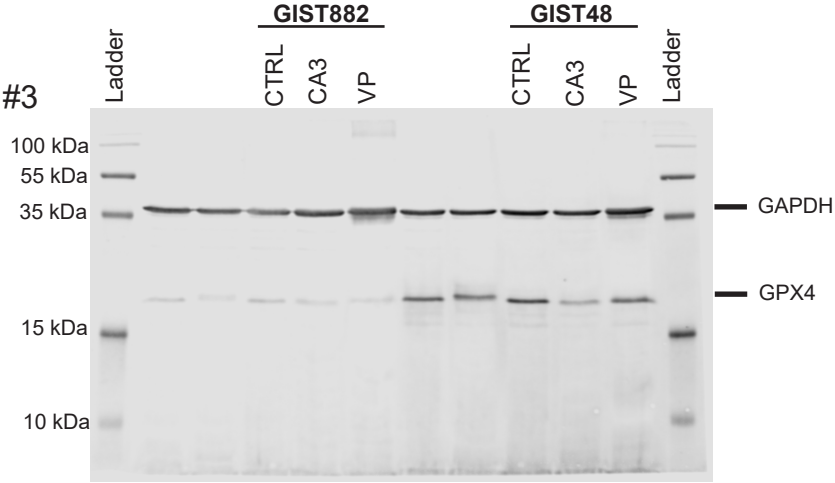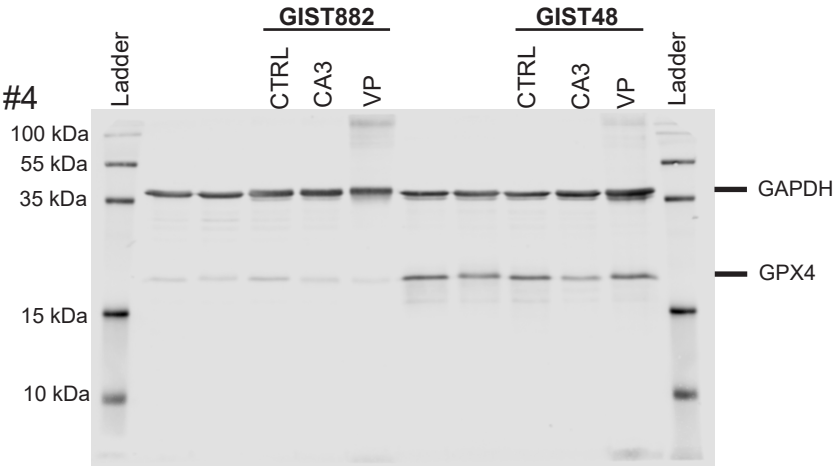

Western Blots : SLC7A11

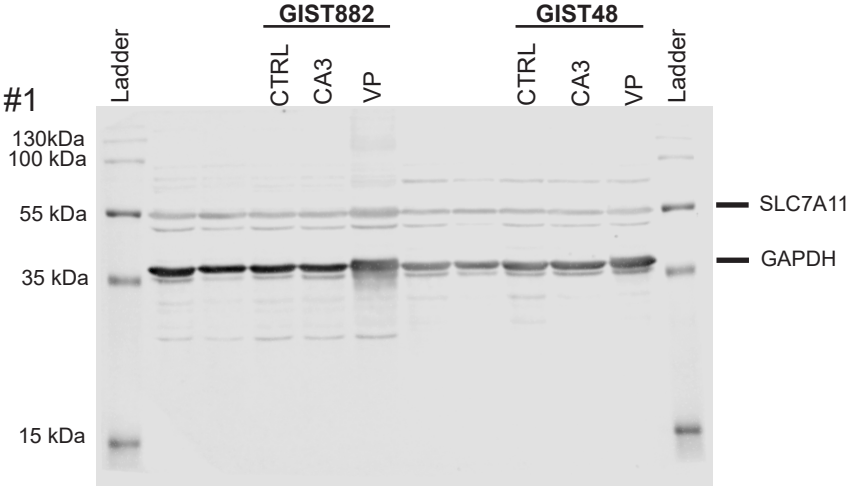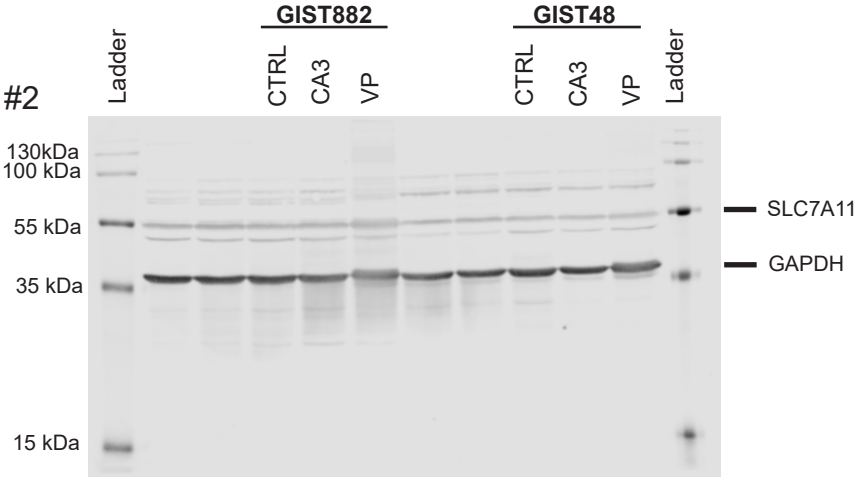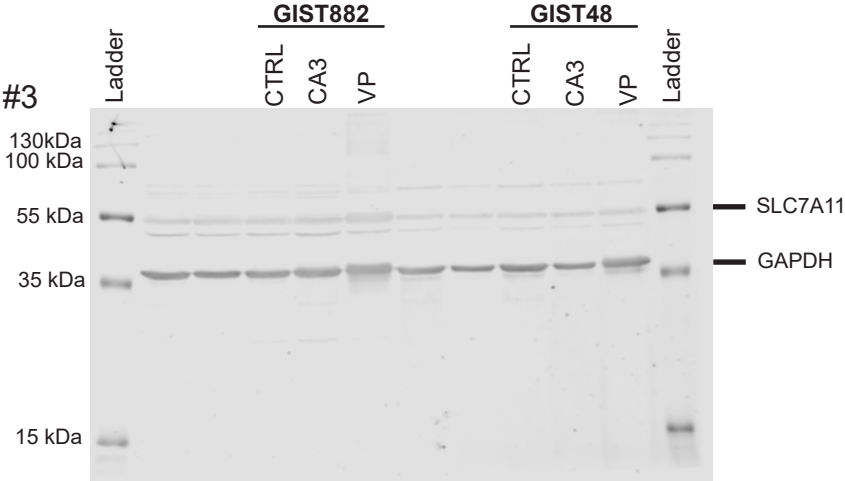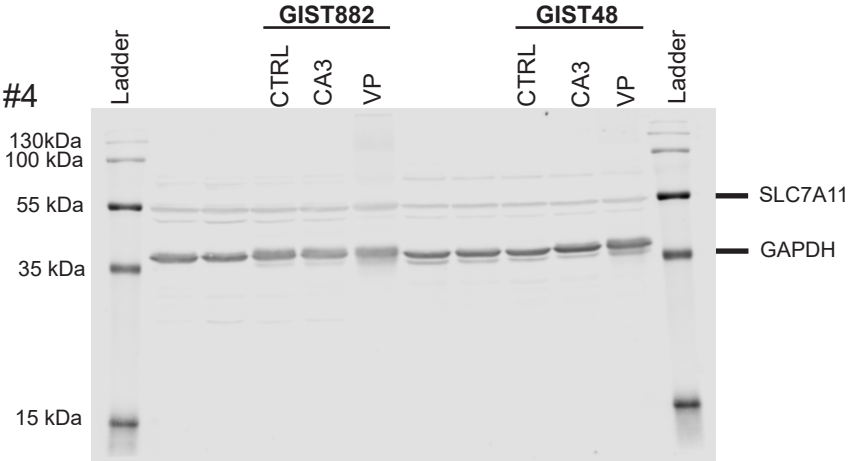

Supplement: Supplementary file 1 [file cancers-14-05050-s001.zip › cancers-1916180 western blot.pdf]
